# Farming system dynamics and sustainability pathways in three districts of Jimma zone, Southwestern Ethiopia

**DOI:** 10.1371/journal.pone.0359541

**Published:** 2026-10-28

**Authors:** Tamiru Chalchisa, Abayineh Amare, Teferi Tolera, Meskelu Daniel, Dessalegn Obsi Gemeda

**Affiliations:** 1 Department of Rural Development and Agricultural Extension, College of Agriculture and Veterinary Medicine, Jimma University, Jimma, Ethiopia; 2 Department of Natural Resource Management, College of Agriculture and Veterinary Medicine, Jimma University, Jimma, Ethiopia; 3 New Era and Development in Civil Engineering Research Group, Scientific Research Center, Al-Ayen Iraqi University (AUIQ), Nasiriyah, Thi-Qar, Iraq; Wallaga University, ETHIOPIA

## Abstract

Smallholder farmers in southwestern Ethiopia face overlapping challenges that trap them in cycles of poverty and land degradation, yet the feedback mechanisms sustaining these traps remain poorly understood. This study addresses the research question what distinct feedback mechanisms perpetuate low productivity across four coexisting farming systems and system-specific leverage points. We used system dynamics modeling, collecting data from 375 households across three purposively selected districts (Dedo, Gera, and Sekoru), complemented by 21 key informant interviews and 8 focus group discussions. Causal loop diagrams (CLDs) were constructed to map feedback cycles within each system. Our results identified four major farming systems with observable disparities across each system. Farm income ranges from 15,691 Ethiopian Birr (ETB) (subsistence, 33% of households) to 46,114 ETB (coffee-honey, 33%). Meanwhile, frequent extension contact varies from 5.7% (subsistence) to 36.1% (wheat-livestock, 19%). Maize-livestock farmers (15%) overgraze at 2.3 TLU/ha (vs. recommended 0.8), mining soil fertility. Coffee-honey farmers face deforestation declined (canopy cover 60% → 25%) and 81% climate impact perception; wheat-livestock farmers have only 8.3% formal credit access. CLDs identified distinct reinforcing traps: the food-labor nexus in subsistence, the soil mining trap in maize-livestock, the input-labor-pest complex in wheat-livestock, and the deforestation-pollination-price triad in coffee-honey. The findings indicate that each farming system is locked into a distinct self-reinforcing cycle, and that system-specific, testable intervention ranging from nutrition-sensitive safety nets for subsistence to climate-smart agroforestry for coffee-honey are required to break these cycles. These findings provide a dynamic, multi-system framework to guide policy design and future empirical evidence for sustainable smallholder agriculture in Ethiopia and similar contexts.

## 1 Background and rationales

A farming system is defined as a complex, integrated set of crop and livestock activities that a household manages based on its available resources, ecological conditions, and livelihood objectives [1,2]. It encompasses not only what is produced but also how production decisions interact with household consumption, labor allocation, market engagement, and environmental management [3]. Farming systems vary widely along several dimensions: the balance between crops and livestock, the degree of market integration (subsistence-oriented versus commercial), the intensity of input use, and the reliance on perennial versus annual crops [4,5].

In Ethiopia, agriculture supports over 80% of the population, yet smallholder farmers face mounting pressures from population growth, land fragmentation, climate variability, and soil degradation [6,7]. These pressures are particularly acute in southwestern Ethiopia, where high rainfall, forested highlands, and diverse elevation zones have given rise to multiple coexisting farming systems: cereal-based subsistence, coffee-honey agroforestry, maize-livestock mixed, and wheat-livestock mixed systems [7]. Unlike studies that examine these systems in isolation, a systems perspective recognizes that they are functionally linked through flows of biomass, nutrients, labor, and capital, as well as through competition for land and water [8]. For example, coffee expansion into forest areas reduces grazing land for livestock-keeping households; conversely, livestock intensification may increase manure availability for cereal farmers. These interdependencies mean that a policy or shock affecting one system inevitably generates ripple effects across others, yet these dynamics remain poorly documented.

A fundamental debate in agricultural development and sustainability science concerns the most appropriate analytical approach for understanding and intervening in smallholder farming systems. On one side of this debate are scholars who advocate for technology-driven, top-down interventions that emphasize the transfer of improved inputs, seeds, and fertilizers to increase productivity [9,10]. This perspective, rooted in neoclassical agricultural economics, assumes that farmers are rational actors who will adopt profitable technologies once constraints such as credit and information are addressed, and that productivity gains will automatically translate into improved welfare and reduced poverty.

Proponents argue that the primary challenge is one of technology diffusion and market development, and that well-designed input subsidy programs, extension services, and credit schemes can catalyze agricultural transformation [6,11]. On the other side of the debate are scholars who advocate for participatory, systems-oriented approaches that emphasize understanding the complex feedback relationships, trade-offs, and contextual factors that shape farming system dynamics [2,3,12]. This perspective, rooted in agroecology and social-ecological systems theory, argues that farming systems are not merely collections of technologies but complex adaptive systems where biophysical, economic, and social variables interact through feedback loops that can either stabilize or destabilize the system over time [13,14]. Proponents contend that without understanding these causal feedback structures, interventions will fail, create unintended consequences, or exacerbate existing problems [15,16]. They argue that the heterogeneity of smallholder contexts, the non-linear nature of system responses, and the presence of poverty traps mean that one-size-fits-all technological solutions are inherently limited.

This study enters this debate by arguing that while both perspectives offer valuable insights, the technology-driven approach has been overemphasized in Ethiopian agricultural policy, while the systems-oriented approach remains underutilized. We contend that the persistent failure of many agricultural interventions in southwestern Ethiopia stems not from a lack of appropriate technologies or from farmer resistance, but from a fundamental misunderstanding of the causal feedback structures that govern each farming system. Without explicitly mapping these structures and identifying their leverage points, policymakers cannot anticipate how interventions will propagate through the system, whether they will generate virtuous cycles or unintended consequences, or which system-specific interventions are most likely to succeed.

Previous research in Jimma Zone and similar Ethiopian highland contexts has examined smallholder farming systems through three distinct but limited approaches, each reflecting different positions in this debate and leaving critical gaps that this study addresses. The first approach examines individual farming systems in isolation, with studies on the agronomy and economics of coffee production [17], the productivity of maize-livestock systems [18], or the food security outcomes of subsistence farming [19]. While valuable, this single-system focus cannot capture cross-system interactions or trade-offs that are critical for understanding system-wide outcomes and designing effective policies. As key informant interviews in this study revealed, farmers do not operate in isolationthe expansion of coffee into forest areas directly reduces grazing land available to livestock keepers, while maize farmers’ use of crop residues for fuel reduces feed availability for neighboring livestock systems.

The second approach uses quantitative impact evaluation methods such as endogenous switching regression and propensity score matching to estimate the productivity or welfare effects of adopting a particular system [11]. These studies correct for selection bias but treat system choice as a discrete, static outcome rather than as a dynamic process embedded in a web of feedback relationships that evolve over time. This static framing is problematic because smallholder farming systems are inherently dynamic: a household’s system choice influences its asset base, soil fertility, and labor availability, which in turn shapes future system choices. Without capturing these feedback dynamics, policymakers cannot anticipate how interventions will unfold over time or whether they will generate unintended consequences.

The third approach consists of qualitative case studies that document farmer perceptions of challenges and adaptation strategies [11,18], but these studies rarely synthesize findings into a formal systems model that can identify leverage points for intervention. While rich in detail, descriptive studies do not specify the causal relationships between variables, distinguish between reinforcing loops that drive virtuous cycles and balancing loops that impose limits, or compare feedback structures across different system types to derive system-specific intervention points. This study argues that the limitations of these three approaches reflect a shared weakness: they do not explicitly model the causal feedback structures that govern farming system dynamics, and therefore cannot resolve the fundamental debate about whether technology-driven or systems-oriented interventions are more appropriate in different contexts.

Consequently, as the critical gap, there is limited study that systematically mapped causal feedback loops across coexisting farming systems in Jimma Zone using formal system dynamics. Specifically, previous work has not: identified how biophysical, economic, and social variables within each system are causally linked, distinguished between reinforcing loops that drive virtuous cycles of improvement and balancing loops that impose limits or create traps or compared feedback structures across different system types to derive system-specific intervention points. Without this dynamic, causal understanding, policymakers lack the ability to anticipate how an intervention targeting one system such as coffee price support, livestock credit, or wheat seed distribution might create unintended consequences or ripple effects in other systems.

This study addresses these gaps by employing a system dynamics modeling approach, specifically causal loop diagramming, to achieve three objectives. First, we empirically identify the four dominant farming systems in Jimma Zone based on household survey data and participatory mapping. Second, we construct causal loop diagrams for each system using qualitative data from focus group discussions, key informant interviews, and field observations, thereby making explicit the causal relationships and feedback loops that drive each system’s behavior over time. Third, we compare these causal structures across systems to identify both common trap mechanisms. By making feedback relationships visible and traceable, this study moves beyond static, single-system analysis to provide a dynamic, multi-system understanding. The resulting causal loop diagrams give a transparent, replicable tool for policy design, enabling decision-makers to anticipate ripple effects and identify high-leverage interventions before implementation.

### 1.1 Conceptual framework of the study

This study is grounded in the social-ecological systems (SES) framework, which conceptualizes agricultural landscapes as complex, adaptive entities where ecological processes and human activities are inextricably linked [13,20]. The SES framework identifies four core subsystems that interact to produce system outcomes: the resource system (land, water, climate), the resource units (crops, livestock, trees), the actors (farming households, their assets and decisions), and the governance system (institutions, policies, extension services) [13]. These subsystems are linked through flows of nutrients, labor, capital, and information, creating feedback loops that can either stabilize or destabilize the system over time [12,14].

While the SES framework provides a valuable starting point, it does not by itself explain how specific variables within each subsystem are causally connected, nor does it identify which feedback loops are most consequential for sustainability. Therefore, this study operationalizes the SES framework by translating each theoretical component into measurable variables collected through household surveys, key informant interviews, and focus group discussions, and then uses these variables to construct causal loop diagrams that explicitly map feedback relationships (see Fig 1).

**Fig 1:**
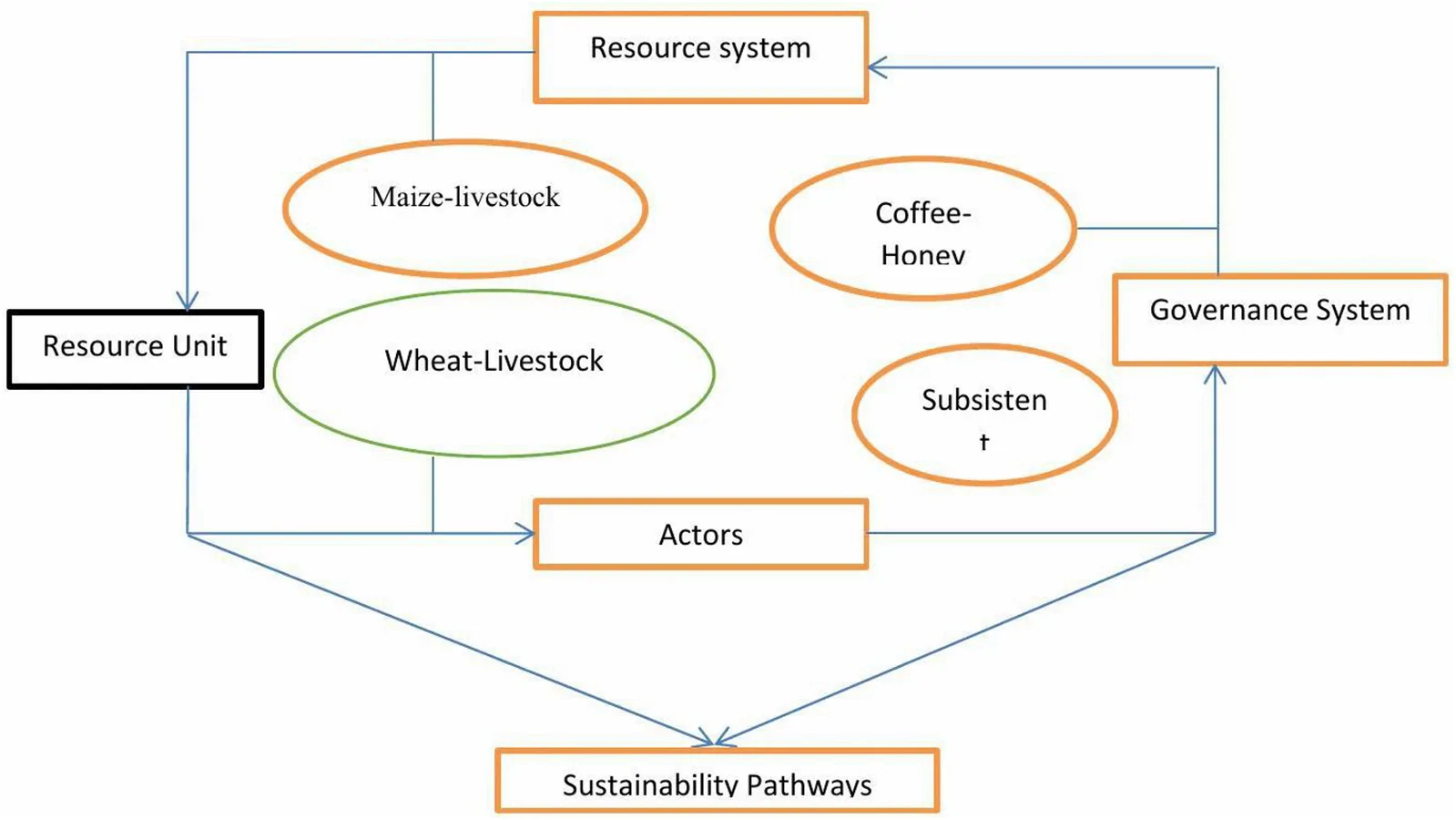
Conceptual framework of the study. **Source: Authors sketch, 2025**.

### 1.2 Operationalization of the framework

Table 1 presents the direct mapping between each SES framework component, the specific variables measured in this study, the data source for each variable, and how each variable appears in the causal loop diagrams.

**Table 1:** Operationalization of the social-ecological systems framework.

SES Component	Theoretical Definition	Measured Variables	Data Source	Role in Causal Loop Diagrams
Resource System	The biophysical environment that provides the foundation for production	Total land holding (ha), number of plots, land certification status, perceived climate change impact	Household survey	Appears as “land availability,” “land degradation,” “climate change impact” as exogenous drivers or balancing loop constraints
Resource Units	The specific crops, livestock, and trees that farmers manage	Maize yield, wheat yield, coffee yield, honey yield, livestock holding, tree cover	Household survey, FGDs on system mapping	Appear as outcome variables (“crop yield,” “honey yield”) and as linking variables (“manure quality,” “feed availability”)
Actors	The farming households who make decisions about resource use	Family size, age, education, farming experience, wealth status (poor/medium/better-off), farm income, off-farm income, labor allocation decisions	Household survey, FGDs on decision-making	Appear as “labor availability,” “financial capacity,” “farmer awareness,” “investment in farming”
Governance System	The formal and informal institutions that shape access and behavior	Extension contact frequency, cooperative membership, credit access, market access, tenure security	Household survey KIIs with development agents and cooperative leaders	Appear as “extension contact,” “cooperative strength,” “credit access,” “market access” as moderators of feedback loops
Interactions	The flows and exchanges between subsystems	Biomass transfer (crop residues used as feed), nutrient cycling (manure applied to fields), labor allocation between crop and livestock, competition for land (coffee expansion vs. grazing), income flows (farm vs. off-farm)	FGDs on resource flows; KIIs on inter-system linkages; household survey on income sources	Form the causal links between variables in CLDs; appear as arrows (e.g., “maize stover → feed availability”)
Outcomes	The system-level results that matter for sustainability	Food security (perceived adequacy), farm productivity (qu/ha), household income (ETB), soil fertility trend (perceived), resilience to shocks (drought, price crash)	Household survey (Section 3.2, Tables 2 and 3); FGDs on challenges and adaptations	Appear as end-point variables in each CLD (e.g., “farm profit,” “household food security,” “soil fertility”)

Source: Adapted from Ostrom [13] and McGinnis and Ostrom [20]; variables derived from field survey, 2025.

## 2 Research methodology

### 2.1 Area description

#### 2.1.1 Geographic locations.

The study was conducted in Jimma Zone, located in the southwestern part of Ethiopia, spanning coordinates 7°15′ to 8°56′ N and 36°00′ to 38°38′ E [21]. Jimma Zone is situated approximately 335 km Southwest of Addis Ababa, the capital of Ethiopia. The zone shares borders with Illubabor Zone to the North, Yam Special Zone to the East, Southwestern Ethiopia Regional State to the South and West.

#### 2.1.2 Climatic region and agro-ecological zones.

Jimma Zone falls within the humid tropical climate region of Ethiopia, characterized by a bimodal rainfall pattern. The area receives an average annual rainfall ranging from 1,200 mm to 2,800 mm, with the main rainy season (kiremt) occurring from June to September and a shorter rainy season (belg) from March to April [22]. Mean annual temperatures vary from 12°C to 26°C, depending on elevation. Based on the traditional Ethiopian agro-ecological classification, the zone has three distinct zones: highlands (Dega) above 2,500 meters above sea level (masl) covering 15% of the area, midlands (Woinadega) between 1,500 and 2,500 masl covering 67% of the area, and lowlands (Kolla) below 1,500 masl covering 18% of the area [22,23]. This agro-ecological diversity directly shapes farming system distributions.

#### 2.1.3 Soil types and fertility status.

The soils of Jimma Zone are predominantly derived from volcanic and basement rock formations. The major soil types include Nitisols (well-drained, deep, fertile red soils) in the midlands and highlands, Vertisols (clay-rich, high water-holding capacity) in the lowland valleys, and Acrisols (highly weathered, low fertility) on steep slopes and degraded areas [24,25]. Nitisols, which cover approximately 60% of the zone, are naturally fertile and support coffee, enset, and maize production. However, continuous cultivation without adequate fallow or organic matter replenishment has led to soil organic carbon decline at rates of 0.5–1.2% annually [26]. Vertisols, covering about 20% of the zone, are challenging for crop production due to poor drainage and hard-setting properties during dry periods, requiring specific management practices such as broad-bed and furrow systems. Acrisols, found on deforested slopes, are highly acidic (pH 4.5–5.5) and nutrient-poor, limiting crop choice primarily to acid-tolerant species such as maize and certain legumes [27]. Soil fertility status varies significantly by farming system: coffee-honey systems, where shade trees maintain organic matter, generally have higher soil organic carbon (2.5–3.5%) compared to subsistence cereal systems (1.0–1.8%) [8].

#### 2.1.4 Land use and land cover.

The total area of Jimma Zone is approximately 1.5 million hectares. The major land use categories include cropland (42%), forest and woodland (28%), settlement (8%), grazing land (15%), and other uses (7%) [22]. However, these land use patterns have been rapidly changing. Historical forest cover, estimated at 60–70% of the zone in the 1970s, has declined to approximately 28% due to coffee plantation expansion, cereal cultivation, and settlement (Kindu et al., 2024). Deforestation rates are highest in the midlands (1.5–2.0% per year), where coffee expansion is most active [27]. The remaining forest is primarily found in protected areas (such as Belete-Gera Forest) and as fragmented patches within coffee agroforestry systems. Grazing land has also declined as cropland expands, increasing pressure on remaining pastures and contributing to overgrazing (livestock density currently averages 2.3 TLU/ha versus recommended 0.8 TLU/ha) [28].

#### 2.1.5 Selected study districts.

The study was conducted in three purposely selected districts Dedo, Gera, and Sekoru; chosen specifically to encompass the full range of agro-ecological zones and farming system diversity within Jimma Zone.

Dedo district is located in the highland zone (2,200–2,800 masl), with average annual rainfall of 1,400–1,800 mm and mean temperature of 12–16°C. Soils are predominantly Nitisols with some Vertisols in valley bottoms. The district is characterized by wheat-livestock mixed systems and enset-based subsistence farming. Landholding sizes average 1.4 ha per household, with significant plot fragmentation.

Gera district is located in the midland zone (1,600–2,200 masl), with average annual rainfall of 1,800–2,400 mm and mean temperature of 18–20°C. Soils are predominantly deep, fertile Nitisols. The district is the core coffee-growing area of Jimma Zone, with coffee-honey agroforestry systems covering the majority of cultivated land. Remaining natural forest patches (e.g., Belete-Gera Forest) provide critical pollinator habitat.

Sekoru district is located in the lowland zone (1,000–1,600 masl), with average annual rainfall of 1,200–1,600 mm and mean temperature of 20–26°C. Soils are a mix of Nitisols on upper slopes and Acrisols and Vertisols in lower areas. The district is characterized by maize-livestock mixed systems and subsistence cereal production (see Fig 2).

**Fig 2:**
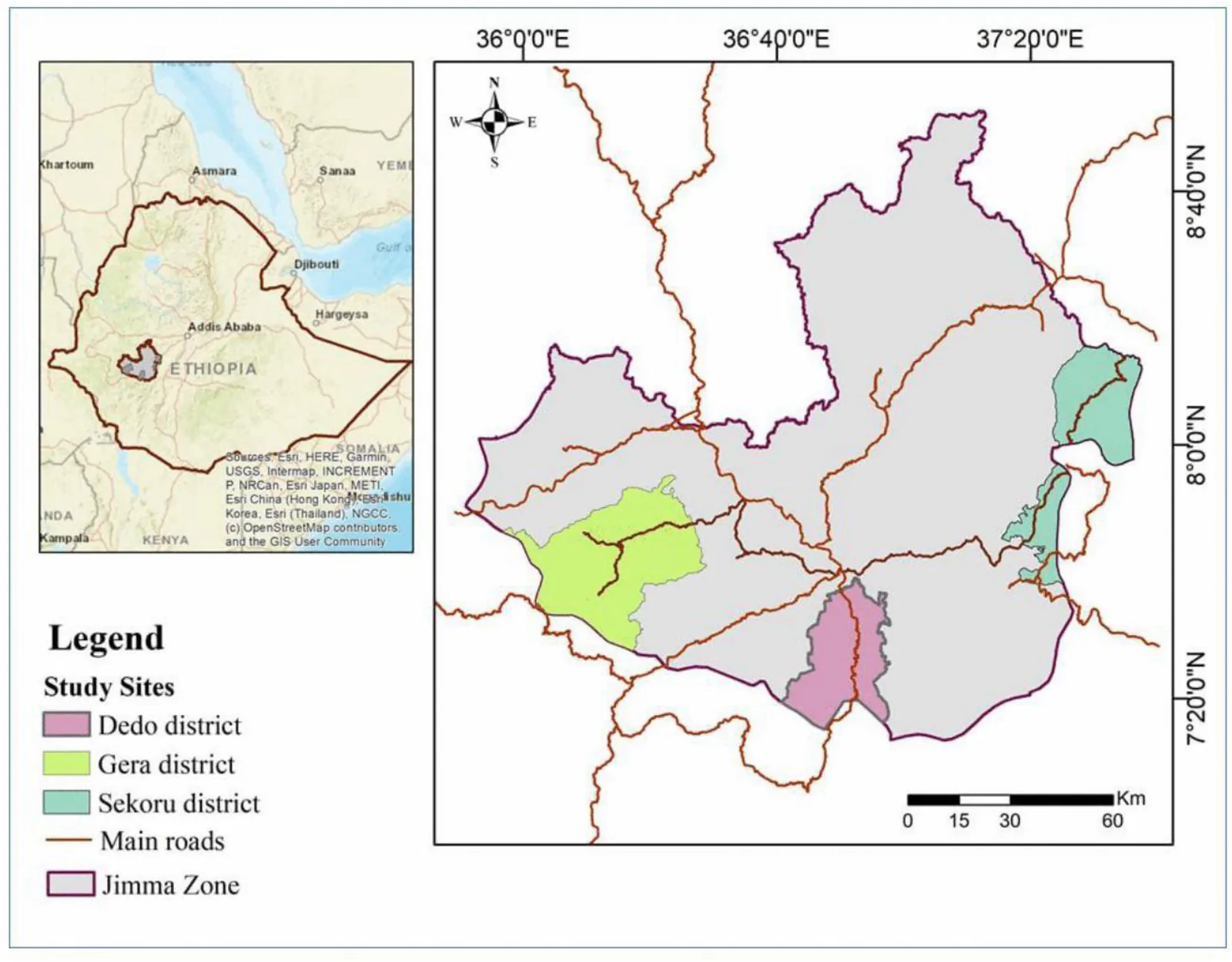
Map of the study area. Source: Created by author using ArcGIS captured from USGS Landsat data (https://earthexplorer.usgs.gov; public domain).

### 2.2 Research design and overall approach

This study employed a convergent mixed-methods design within a system dynamics framework. A qualitative-dominant approach was used to capture the complex, nuanced relationships and feedback mechanisms within farming systems, while quantitative data provided structural and statistical context to validate and quantify key variables. The research was guided by a phenomenological perspective to understand farmers’ lived experiences and systems thinking to analyze interlinkages between ecological, economic, and social components.

### 2.3 Sampling design and household selection

A multi-stage stratified random sampling technique was employed to ensure representation across the three distinct agro-ecological zones of Jimma Zone: highland (Dega), midland (Woinadega), and lowland (Kolla). Since farming systems are closely associated with these agro-ecological zones all major farming systems would be captured. The sampling proceeded in four stages.

**Stage 1: District selection**

Three districts (woredas) Dedo, Gera, and Sekoru district were purposively selected from the 21 districts of Jimma Zone to represent the three agro-ecological zones. As described in Section 2.1, Dedo district represents the highland zone (Dega), 2,200–2,800 masl, where wheat-livestock and enset-based subsistence systems predominate. Gera district represents the midland zone (Woinadega), 1,600–2,200 masl, where coffee-honey systems predominate. Sekoru district represents the lowland zone (Kolla), 1,000–1,600 masl, where maize-livestock and cereal-based subsistence systems predominate. This purposive district selection ensured that the full range of agro-ecological variation and thus the full range of farming systems was captured at the highest spatial level.

**Stage 2: Kebele selection**

Within each selected district, kebeles are not uniformly distributed across elevation. Each district spans a range of elevations within its primary agro-ecological zone. Therefore, within each district, kebeles were stratified by elevation to capture variation within the zone. In Dedo district (highland zone), three kebeles were purposively selected from different elevation levels within the highland range (2,200−2,500 masl, 2,500−2,700 masl, and above 2,700 masl) to capture variation in wheat and enset system intensity. In Gera district (midland zone), three kebeles were purposively selected from different elevation levels within the midland range (1,600−1,800 masl, 1,800−2,000 masl, and 2,000−2,200 masl) to capture variation in coffee agroforestry intensity and forest cover. In Sekoru district (lowland zone), three kebeles were purposively selected from different elevation levels within the lowland range (1,000−1,200 masl, 1,200−1,400 masl, and 1,400−1,600 masl) to capture variation in maize intensity and soil types (Nitisols on upper slopes, Acrisols on mid-slopes, Vertisols in valley bottoms).

A total of nine kebeles (three per district) were selected. Within each elevation stratum within a district, the specific kebele was selected purposively based on accessibility, representativeness of the farming system, and availability of household lists from kebele administrative offices. Table 2 summarizes the kebele selection criteria by agro-ecological zone and elevation.

**Table 2:** Selection of districts and kebeles by agro-ecological zone and elevation.

District	Agro-ecological zone	Elevation range (masl)	Number of kebeles	Elevation strata within zone (masl)	Selection criteria within strata
Dedo	Highland *(Dega)*	2,200−2,800	Ilala, Sito and Sheger	2,200−2,500; 2,500−2,700; > 2,700	Wheat intensity; slope gradient
Gera	Midland (*Woinadega)*	1,600−2,200	Sedi Loya, Ganji Chala and Wanji Kersa	1,600-1,800; 1,800-2,000; 2,000-2,200	Forest cover gradient; distance to market
Sekoru	Lowland *(Kolla)*	1,000-1,600	Bore, Chala and Gengeleta	1,000−1,200; 1,200−1,400; 1,400−1,600	Soil type (Nitisol/Acrisol/Vertisol); maize intensity

**Stage 3: Household selection within kebeles**

Within each selected kebele, a complete household list was obtained from the kebele administrative office. Households were then stratified by wealth status (poor, medium, and better-off) based on kebele records and local development agent knowledge. This wealth stratification was applied to ensure that households from all economic backgrounds were represented within each agro-ecological zone, as wealth status is known to influence farming system participation and productivity outcomes. From each wealth stratum within each kebele, households were then selected using simple random sampling (lottery method).

**Stage 4: Sample size calculation and allocation**

The total sample size of 375 households was calculated using the Kothari formula [29]:


$$\begin{array}{c} n=\frac{z^{\text{2}}*p*q*N}{e^{\text{2}}(\text{N}-\text{1})+{\text{z}}^{\text{2}}*p*q}\approx \text{ 375} \end{array}$$

Where; n = minimum returned sample size

N= Population size (14,200 household heads)

e= level of precision/ adjust margin of error= 5%

Z refers to the critical value of the standard normal distribution at a 95% confidence level, which is 1.96

p = estimated proportion (0.5)

**q** Is calculated as 1-**p**.

The 375 households were allocated proportionally to each agro-ecological zone based on the estimated number of farming households in each zone (probability proportional to size). Within each zone, households were further allocated across the three kebeles (elevation strata) and across wealth strata proportionally.

To further justify the sample size, a post-hoc power analysis was conducted using *G*Power version 3.1.9.7 [30] for two-independent-means t-tests, which are the primary inferential tests for comparing continuous outcomes between farming systems. Assuming a moderate effect size (Cohen’s d = 0.40), a significance level of α = 0.05 (two-tailed), and a desired power of 0.80, the required sample per group is approximately 100. Our smallest farming system group (maize-livestock, n = 56) achieves a power of 0.72, while the larger groups (subsistence, n = 122; coffee-honey, n = 125) exceed 0.85. Given the exploratory nature of this mixed-methods study and the triangulation of qualitative evidence, a power of 0.72 for the smallest group is considered acceptable [31]. Moreover, all comparisons between larger groups are sufficiently powered (>0.85).

### 2.4 Methods of data collection

Data were collected through three primary, iterative phases between January and June 2025. Qualitative data were gathered through focus group discussions and key informant interviews. Eight focus group discussions were conducted, with two discussions per identified farming system typology, subsistence, coffee-honey, maize-livestock, and wheat-livestock, distributed across the three districts to capture agro-ecological variation. For each FGD, eight to ten participants were purposively selected by local development agents and kebele administrators to ensure representation of experienced farmers, younger farmers, different wealth categories, gender, and full dependence on the target farming system.

Each discussion lasted two to two-and-a-half hours and was facilitated by two PhD-level researchers (one male, one female) who are fluent in Afan Oromo and have previous experience working in the study districts. They used a semi-structured guide that focused on system mapping, causal relationship elicitation, feedback loop identification, and challenges and solutions narratives. All FGDs were audio-recorded, transcribed verbatim, and translated into English. Additionally, twenty-one key informant interviews were held with district agricultural officers, development agents, local elders, and cooperative leaders to explore institutional linkages, policy effects, and historical trends.

To ensure rigor and transparency in qualitative sampling, the number of focus group discussions (n = 8) and key informant interviews (n = 21) was determined using a prospective data saturation protocol adapted from Guest et al. [32] and Hennink and Kaiser [33]. Saturation was defined as the point at which three consecutive data collection events (e.g., one FGD plus two KIIs) yielded no new variables, causal relationships, or emergent themes beyond those already identified.

Data collection was organized into three sequential batches: Batch 1 (FGDs #1–3, KIIs #1–7), Batch 2 (FGDs #4–6, KIIs #8–14), and Batch 3 (FGDs #7–8, KIIs #15–21). After each batch, the research team conducted debriefing sessions to code new variables and track thematic emergence. Saturation was achieved after Batch 2 for FGDs (42 cumulative variables; only 5 new, low-frequency variables emerged in Batch 3) and after Batch 2 for KIIs (38 cumulative variables; only 6 new variables emerged in Batch 3). Participant selection for both FGDs and KIIs followed a purposive criterion-based sampling strategy to ensure maximum variation within each farming system.

For FGDs, selection criteria included: (a) full-time farming experience in the target system for ≥5 years (minimum 50% of participants per FGD); (b) age diversity (at least 2 participants ≤35 years and 2 > 35 years); (c) gender (minimum 1 female participant per FGD, where present); (d) wealth category (at least 1 participant each from poor, medium, and better-off categories based on kebele wealth ranking); and (e) landholding size (at least 1 participant each from small <1 ha, medium 1–2 ha, and large >2 ha categories). For KIIs, selection criteria included institutional role (district agricultural officers, development agents, kebele administrators, cooperative leaders, and local elders) and minimum 5 years of experience in their respective roles.

Quantitative data were collected through a structured household survey administered to 375 randomly selected households from nine kebeles across the three districts. Kothari sample size determination formula was used to reach on the number of representative sample from the study populations. In this study, a 5% (0.05) precision level with a 95% confidence level was used. Based on the formula 375 sample respondents were selected through employing simple random sampling. The sample size of each kebeles is determined by probability to proportional sample size. The questionnaire was pre-tested on 30 households (10 per district) for clarity, relevance, and cultural appropriateness, and revisions were made accordingly.

The survey collected data on socio-demographics, farm characteristics, production data, input-output economics, and perceptions regarding soil fertility, constraints, and climate change impacts. This data provided the quantitative variables that were later linked to qualitative variables in the causal loop diagrams. Field observations across all study kebeles documented farming practices, land use patterns, soil conditions, and livestock management, providing contextual grounding for interview and survey data. A completed checklist of compliance with the CROSS and COREQ guidelines is provided in Supplementary S4 File.

### 2.5 System dynamics modeling: Causal loop diagram construction and validation

The construction of causal loop diagrams (CLDs) followed a systematic, four-phase protocol adapted from Sterman [34] and validated participatory modeling approaches for smallholder systems [15,16]. Each phase is described below with explicit procedures, coding rules, and validation criteria to ensure reproducibility.

**Phase 1: Variable identification and extraction from qualitative data**

**Step 1.1: Transcription and initial coding**

All focus group discussions (n = 8) and key informant interviews (n = 21) were audio-recorded, transcribed verbatim in Afan Oromo, and then translated into English by two bilingual research assistants. Translations were cross-checked for consistency by a third researcher. Transcribed texts were imported into NVivo 14 qualitative data analysis software.

**Step 1.2: Open coding to identify system variables**

Two researchers independently performed open coding on 30% of the transcripts (selected randomly) to identify variables mentioned by participants as important elements of their farming system. A variable was defined as any noun or noun phrase that participants described as changing over time, being influenced by other factors, or influencing outcomes. Examples included “soil fertility,” “labor availability,” “coffee yield,” “market price,” and “forest cover.” Each researcher produced an initial code list.

**Step 1.3: Consensus variable list**

The two researchers met to compare their independent code lists. Variables with >80% agreement on presence and labeling were retained. Disagreements were resolved through discussion with a third researcher. This process yielded an initial variable list of 47 unique variables across all four farming systems.

**Step 1.4: Variable inclusion criteria**

Variables were retained for CLD construction only if they met all three of the following inclusion criteria indicated in Table 3.

**Table 3:** Variable inclusion criteria.

Criterion	Operational Definition	Example
C1: Mention frequency	Variable mentioned by at least 5 distinct participants (across at least two FGDs or KIIs)	“Soil fertility” mentioned in 7/8 FGDs → included. “Specific fertilizer brand” mentioned only once → excluded.
C2: System relevance	Variable has at least one incoming or outgoing causal link reported by participants	“Rainfall” has causal link to “crop yield” → included. “Name of local market” has no causal link → excluded.
C3: Policy relevance	Variable is potentially modifiable by policy or management (excluding purely exogenous factors beyond human influence, except climate)	“Extension contact” is modifiable → included. “Global coffee price” is exogenous but retained due to policy relevance (risk management).

**Step 1.5: Variable grouping by system**

Variables were assigned to one of the four farming systems (subsistence, coffee-honey, maize-livestock, wheat-livestock) based on the primary system in which they were mentioned. Variables that appeared across multiple systems (e.g., “climate change impact,” “credit access”) were retained in each system’s CLD to allow cross-system comparison.

**Phase 2: Causal relationship extraction or Link identification**

**Step 2.1: Identifying causal statements**

From the coded transcripts, researchers extracted all sentences or statement sequences that expressed a causal relationship between two variables. A causal statement was defined as any expression containing explicit causal language (e.g., “leads to,” “causes,” “results in,” “due to,” “because of”) or implicit causal sequence (e.g., “when X increases, Y decreases”). Each causal statement was entered into a structured extraction table with the following fields (Table 4):

**Table 4:** Causal statements.

Sn	Field	Example
1	Statement ID	S-023
2	Source	FGD #3 (Maize-livestock), Participant 7
3	Original quote (translated)	“When we have more manure, the maize yields are much higher”
4	Independent variable	Manure availability
5	Dependent variable	Maize yield
6	Direction of effect	Positive (+)
7	Strength (as reported)	Strong (participant used “much higher”)
8	Time lag (as reported)	Same season

**Step 2.2: Coding rules for causal relationships**

The following rules (Table 5) were applied to code each extracted causal statement:

**Table 5:** Coding rules.

Rule	Description	Application
**R1: Direction**	Effect is coded as positive (+) if an increase in the independent variable leads to an increase in the dependent variable, or a decrease leads to a decrease. Effect is coded as negative (-) if an increase leads to a decrease, or a decrease leads to an increase.	“More manure → higher yield” = (+). “More deforestation → less honey” = (-).
**R2: Confidence**	Link is coded as “high confidence” if at least 5 participants from at least 2 different data sources (e.g., FGD and KII) reported the same direction of effect. “Medium confidence” if 3–4 participants or only one data source. “Low confidence” if 1–2 participants.	Only high and medium confidence links were retained for CLD construction (see Phase 3).
**R3: Directness**	Only direct causal links (no intermediate variables) were coded. If a participant described an indirect chain (X → Y → Z), each direct link (X → Y and Y → Z) was coded separately.	“Drought reduces feed, which weakens livestock” → coded as two links: (drought → feed availability) and (feed availability → livestock health).
**R4: Bidirectionality**	If participants reported that the relationship operates in both directions (X → Y and Y → X), two separate links were coded.	“When we have more income, we buy more animals. And when we have more animals, we have more income from sales.” → coded as (income → livestock) and (livestock → income).

**Step 2.3: Link aggregation and filtering**

After extracting all causal statements, links were aggregated by (independent variable, dependent variable, direction). A link was retained for CLD construction if it met both of the following retention criteria: Retention criterion A: The link was reported by at least 3 distinct participants. Retention criterion B: The link appeared in at least 2 separate data collection events (e.g., one FGD and one KII, or two different FGDs). Links that did not meet these criteria were documented in a supplementary file but excluded from the main CLDs.

**Phase 3: Causal loop diagram construction**

**Step 3.1: Draft CLD creation using Vensim**

Using the retained variables and causal links for each farming system, draft CLDs were constructed in Vensim PLE version 10.4 following these construction rules (Table 6)

**Table 6:** Casual loop constriction rules.

Construction Rule	Description
**Variables**	Represented as boxes or words (not circles, per Vensim convention for CLDs)
**Causal links**	Represented as arrows with polarity marked: (+) for positive effect, (-) for negative effect
**Loop identification**	Loops were traced manually and labeled as Reinforcing (R) if the product of polarities around the loop is positive (self-amplifying) or Balancing (B) if the product is negative (self-correcting)
**Loop naming**	Each loop was given a descriptive name (e.g., “Food-labor nexus”) based on the dominant variable in the loop and participant narratives

**Step 3.2: Loop polarity calculation**

For each identified loop, polarity was calculated as the product of all link polarities around the loop. A loop was labeled Reinforcing (R) if the product was positive (odd number of negative links = negative product as indicated below.

- Even number of negative links = positive product = Reinforcing loop (R)
- Odd number of negative links = negative product = Balancing loop (B)

*Example: A loop with three links: (+) × (+) × (-) = (-) → Balancing loop.*

**Step 3.3: Cross-system comparison protocol**

After individual system CLDs were constructed, a cross-system comparison was conducted following a structured protocol:

1. Variable presence matrix: For each variable in each system’s CLD, we recorded whether the variable appeared (1) or did not appear (0). Variables appearing in at least three of the four systems were designated “common challenge variables.”
2. Shared loop identification**:** Loops with identical or analogous structures across systems were identified (e.g., the “low income → low investment → low yield → low income” loop appeared in subsistence, maize-livestock, and wheat-livestock systems, with different specific variables).
3. System-specific loop cataloging: Loops unique to a single system were documented as “system-specific constraints” (e.g., the pollination loop in coffee-honey).

**Phase 4: Participatory model validation**

**Step 4.1: Member checking with original participants**

A one-day validation workshop was convened with a subset of original FGD participants (n = 24; 6 from each farming system). The workshop followed a structured validation protocol indicated in Table 7 and supplementary S3 File.

**Table 7:** Member validations.

Validation Activity	Procedure	Success Criterion
**Variable verification**	Draft CLDs were printed on large-format paper (A0 size). Participants were asked: “Are all the important parts of your farming system shown here? Is anything missing?”	≥80% of participants agreed that the variable set was complete. Missing variables suggested by ≥3 participants were added.
**Link verification**	For each arrow in the CLD, participants were asked: “Does X really affect Y in the direction shown? Can you give an example?”	Links with <70% participant agreement were flagged for re-examination. Disconfirmed links were removed.
**Loop verification**	Participants were asked: “Do you see this cycle happening in your farm? Can you describe a time when you experienced this?”	Loops that could not be illustrated with a concrete participant example were re-examined or removed.
**Loop naming**	Participants were invited to name each loop in their own words (Afan Oromo). Names were recorded and translated.	At least one participant-generated name per loop; names were incorporated into final CLD labels.

**Step 4.2: Expert validation**

Following participant validation, the revised CLDs were presented to a panel of domain experts (n = 5): two agricultural economists, one system dynamics modeler, and two senior agricultural researchers with experience in Ethiopian farming systems. Experts were asked to evaluate: Theoretical consistency: Do the causal relationships align with established agricultural systems literature? Logical completeness: Are there missing variables or links that standard theory would predict? Practical utility: Would these CLDs be useful for policy design? Expert feedback was incorporated in a second round of revisions. The final CLDs achieved consensus agreement from all five experts.

**Step 4.3: Documentation of rejected and modified elements**

All variables, links, and loops that were proposed during Phases 1–3 but subsequently rejected or modified during validation were documented in a model audit trail (supplementary file). For each rejected element, the reason for rejection (e.g., “participants reported no causal link,” “link contradicted by expert review,” “variable mentioned by only 2 participants”) was recorded. This audit trail ensures transparency and reproducibility.

#### 2.5. 1 Data triangulation protocol.

To operationalize the convergent mixed-methods design and ensure transparent integration of qualitative and quantitative data, this study employs a systematic triangulation matrix framework (adapted from Farmer et al., [35]; O’Cathain et al., [36]). The matrix explicitly maps each variable and causal relationship in the final causal loop diagrams to two sources of evidence: (1) qualitative evidence from focus group discussions and key informant interviews, recorded as specific participant quotes and the number of independent mentions across data sources; and (2) quantitative indicators from the household survey, expressed as descriptive statistics (e.g., percentages, means) or bivariate relationships (e.g., t-test results, correlation coefficients). For each mapped element, the matrix classifies convergence status as “full convergence” (qualitative and quantitative evidence agree in direction and magnitude), “partial convergence” (one source supports the relationship while the other is neutral or weakly supportive), or “divergence” (sources contradict). Divergent cases are flagged for re-examination of original transcripts and survey data; if divergence persists after re-analysis, the variable or link is excluded from the final CLD or retained with a documented caveat. This triangulation protocol ensures that every causal link in the final models is supported by at least two independent forms of evidence, thereby strengthening the validity, transparency, and reproducibility of the system dynamics analysis.

The complete triangulation matrix, containing all 103 variables and causal links with their qualitative and quantitative evidence sources, convergence classifications, and resolution actions, is provided as Supplementary S1 File. A summary of triangulation outcomes by farming system is presented in Supplementary S2 File. Across all systems, 81% of elements achieved full convergence, 12% partial convergence, and 8% were excluded due to divergence, resulting in an overall retention rate of 92%.

### 2.6 Ethical considerations

All study participants provided informed consent prior to their involvement in focus group discussions, key informant interviews, household surveys, and the validation workshop. Participants received clear information about the study’s objectives, methods, and their right to withdraw at any time without consequence. Data were managed in compliance with relevant data protection regulations, and confidentiality and anonymity were maintained throughout the research process.

The study protocol was reviewed and approved by the Institutional Review Board (IRB) of Jimma University College of Agriculture and Veterinary Medicine (approval date: 01/01/2025). Participant recruitment and data collection were conducted from 05/01/2025–20/05/2025, following the full grant of ethical approval.

### 2.7 Field permission

Prior to data collection, informed consent was obtained from all participants, including key informants, focus group discussants, and surveyed households. Furthermore, necessary permissions for fieldwork and community engagement were secured from the relevant local administrative authorities (woreda and kebele offices) in Dedo, Gera, and Sekoru districts. Participants were assured of confidentiality and their right to withdraw from the study at any time without consequence

## 3 Result

### 3.1. Socioeconomic characteristics of sampled respondents

Table 8 shows the key characteristics and livelihoods of 375 sampled farmers. The overwhelming majority (98.4%) of respondents rely on farming as their primary occupation, with only minimal engagement in labor (1.1%) or other activities (0.5%). This near-total dependence on agriculture as both primary and often sole occupation indicates the vulnerability of these households to agricultural risks and market fluctuations. When a single sector dominates household livelihoods, any shock to that sector whether climate-related, economic, or ecological has disproportionate impacts on household welfare. This finding is consistent with national patterns in Ethiopia, where agriculture employs approximately 70–80% of the population [6], but the 98.4% figure in this study exceeds the national average, suggesting that the study area has particularly limited non-farm employment opportunities. This lack of livelihood diversification has important implications for intervention design: programs that focus solely on agricultural productivity improvements may fail to build resilience if they do not also address off-farm income generation.

**Table 8:** Socio-demographic characteristics of the sampled respondents (Categorical).

Variables		Frequency	Percent
Primary Occupation	Farming	369	98.4
	Laborer	4	1.1
	Other	2	.5
Wealth status	Medium	250	66.7
	Poor	84	22.4
	Relatively Better-off	41	10.9
Number of Plot of land owned	Five or above	19	5.1
	Four	43	11.5
	None	3	.8
	One	121	32.3
	Three	83	22.1
	Two	106	28.3

Regarding wealth status, most farmers (66.7%) fall into a “medium” category, while 22.4% are classified as “poor” and only 10.9% as “relatively better-off.” This distribution indicates limited economic diversity within the community and suggests a relatively egalitarian but uniformly low-resource context. The predominance of medium and poor households means that most farmers operate with minimal financial buffers against shocks, and that poverty is not limited to a small minority but is a widespread condition affecting the majority of households. This has implications for the types of interventions that are feasible: capital-intensive technologies or programs requiring significant upfront investment may be inaccessible to the majority of farmers without substantial subsidies or credit. The wealth distribution also suggests that poverty reduction programs targeting the “poor” category alone would leave the majority of “medium” households still vulnerable to falling into poverty following a shock, indicating the need for broad-based rather than narrowly targeted interventions.

Land ownership patterns show that most farmers operate on small-scale, fragmented holdings. About 32.3% own just one plot, 28.3% own two plots, and 22.1% own three plots, while only 5.1% possess five or more plots. A negligible 0.8% owns no land at all, possibly working as laborers. This fragmentation pattern reflects the combined effects of population pressure, inheritance practices that divide land among children, and the limited land market in rural Ethiopia. Land fragmentation has significant implications for agricultural productivity: smaller plots are less efficient to cultivate, reduce opportunities for economies of scale, increase labor requirements for traveling between plots, and complicate the adoption of improved technologies. The finding that the majority of households operate only one to three plots suggests that land consolidation whether through land markets, cooperative farming, or collective action could be an important pathway for improving productivity, though the political and social sensitivities around land in Ethiopia make this a complex policy area.

As indicated in Table 9, farming experience among the respondents varies widely, from 0 to 60 years, with a mean of 23.96 years and a standard deviation of 12.447, reflecting diverse levels of agricultural expertise. This wide range means that policy and extension programs must cater to both experienced farmers who may have extensive local knowledge but limited formal education, and younger farmers who may be more open to innovation but have less practical experience. The mean experience of nearly 24 years suggests that the average farmer has substantial practical knowledge of local conditions, which extension programs should leverage through participatory approaches rather than replacing with external knowledge.

**Table 9:** Socio-demographic characteristics of the sampled respondents (Continuous).

	Minimum	Maximum	Mean	Std. Deviation
Age	20	80	43.23	12.267
Farming Experience	0	60	23.96	12.447
Total land holding in ha	.0000	10.0000	1.863212	1.4010565

Land holdings range from 0 to 10 hectares, with an average of 1.86 hectares and a standard deviation of 1.40, highlighting substantial disparities in land ownership. This suggests a highly unequal distribution, where some farmers possess relatively large plots (up to 10 ha), while others may be landless or have minimal holdings. The coefficient of variation (standard deviation/mean = 0.75) indicates considerable inequality in land distribution, which is a key determinant of wealth and welfare in agrarian societies. Such disparities align with smallholder farming systems, which dominate Ethiopian agriculture, where smallholders typically cultivate plots of less than 2 hectares, relying on subsistence farming with limited mechanization.

The mean landholding in this sample (1.86 ha) closely matches Ethiopia’s national average of 1.0–1.5 hectares, as reported by the Central Statistical Agency (CSA) and World Bank studies. However, the upper range (10 ha) indicates the presence of larger, possibly commercial or semi-commercial farms, which are less common but emerging in the area. This bimodal distribution with most households operating very small plots and a few operating substantially larger plots has important implications for intervention design: policies and programs that are designed for the average farm size may not be appropriate for either the very small or the very large farms, suggesting the need for differentiated approaches.

### 3.2 Farming system typologies

In the study area, four primary farming system typologies were identified: subsistence farming, coffee-honey mixed farming, maize-livestock mixed farming, and wheat-livestock mixed farming (Fig 3). Subsistence farming is widely practiced, followed by coffee-honey mixed farming system, while the maize-livestock and wheat-livestock mixed farming systems follow. The dominance of subsistence farming (32.5%) and coffee-honey systems (33.3%) together accounting for nearly two-thirds of households reflects the agro-ecological conditions of the study area, where the midland and highland zones are suitable for coffee production and where resource-constrained households resort to subsistence strategies.

**Fig 3:**
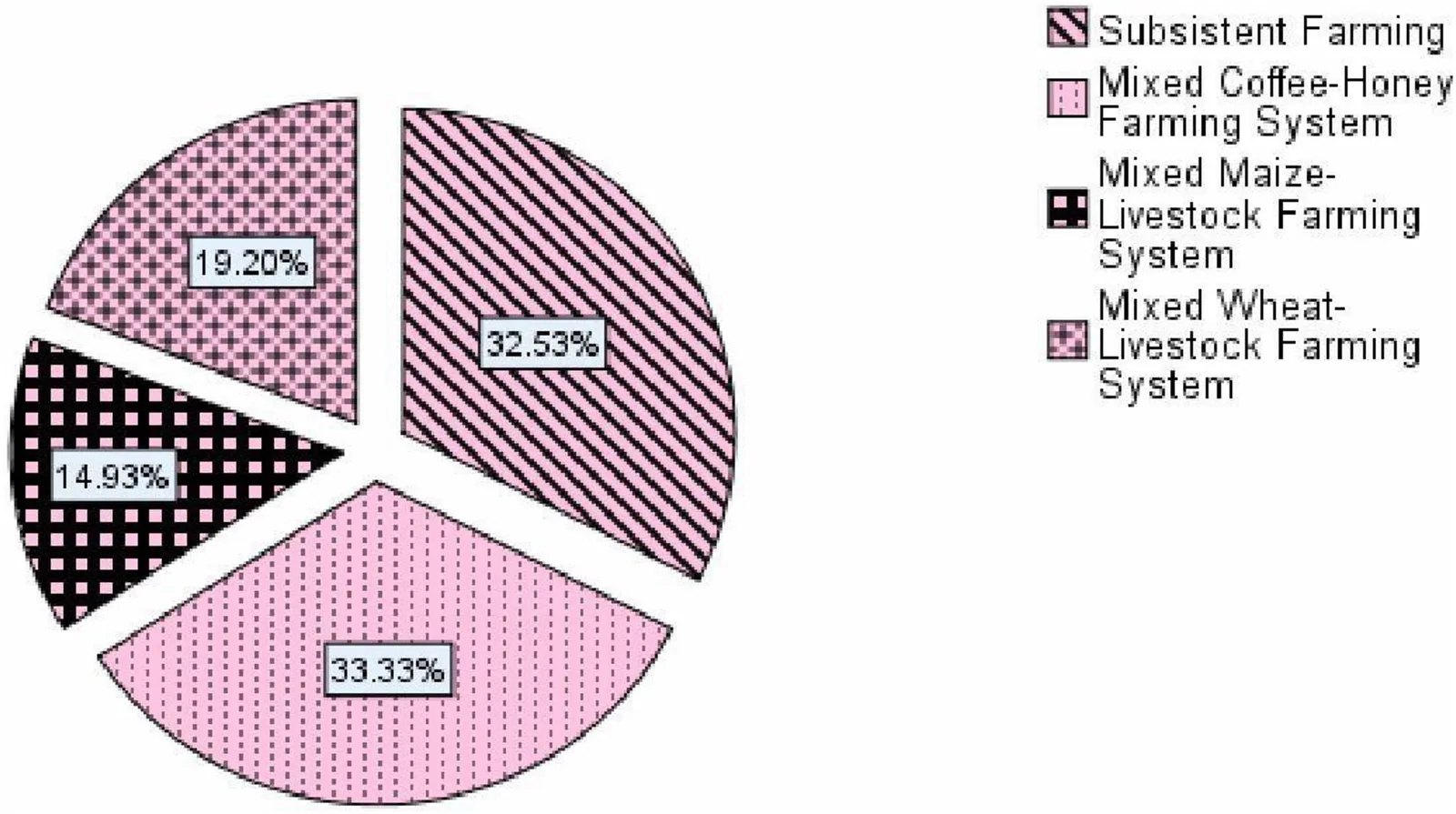
Major farming system typologies. **Source: Field survey, 2025**.

The relatively smaller proportions of maize-livestock (14.9%) and wheat-livestock (19.2%) systems reflect the more specialized nature of these systems, which require specific agro-ecological conditions and access to inputs and markets. The coexistence of these four systems within the same landscape creates both opportunities and challenges: opportunities for complementarity through nutrient flows, labor sharing, and income diversification; challenges through competition for land, water, and biomass resources. This diversity also means that policy interventions designed for one system may have unintended consequences on others, highlighting the need for landscape-level planning. The findings derived from the causal loop diagram are presented below, with detailed descriptions provided in the subsequent sections.

### 3.3 Causal loop diagrams: Empirical findings

#### 3.3.1 Subsistent farming system.

The subsistence system is dominated by a reinforcing poverty trap (R1) (Table 10 and Fig 4). Low financial capacity prevents investment in improved seeds, fertilizer, or equipment, leading to stagnant crop productivity. This loop is particularly difficult to break because it involves both economic (income → investment) and biophysical (food → labor) constraints, suggesting that neither input subsidies alone nor nutrition interventions alone will be sufficient. The food-labor nexus is the critical mechanism through which poverty reproduces itself: households experiencing chronic food insecurity lack the physical capacity to perform the labor required to increase production, even when inputs are available. This finding has profound implications for intervention design: it suggests that the sequencing of interventions matters, with food security and nutrition needing to precede or accompany input provision. This challenges the common practice in agricultural development programs of focusing primarily on input provision without addressing the underlying labor constraints.

**Table 10:** Summary of causal loops in the subsistence farming system.

Loop ID	Type	Key Variables	Core Dynamic (One Sentence)	Policy Relevance
R1	Reinforcing (+)	Low financial capacity → Low technology adoption → Low productivity → Food insecurity → Reduced labor → Low financial capacity	Poverty trap where lack of income prevents investment, keeping yields low and labor weak	High
R2	Reinforcing (+)	Low education → Weak extension contact → Low technology adoption → Low productivity	Low literacy limits ability to access and benefit from extension services	Medium
B1	Balancing (-)	Land scarcity → Land degradation → Lower yields → More land needed	Land degradation reduces productivity, forcing expansion onto marginal lands	High
B2	Balancing (-)	market access → Low output prices → Low income → Low investment	Poor market access prevents fair prices, reducing incentive to produce surplus	High

**Fig 4:**
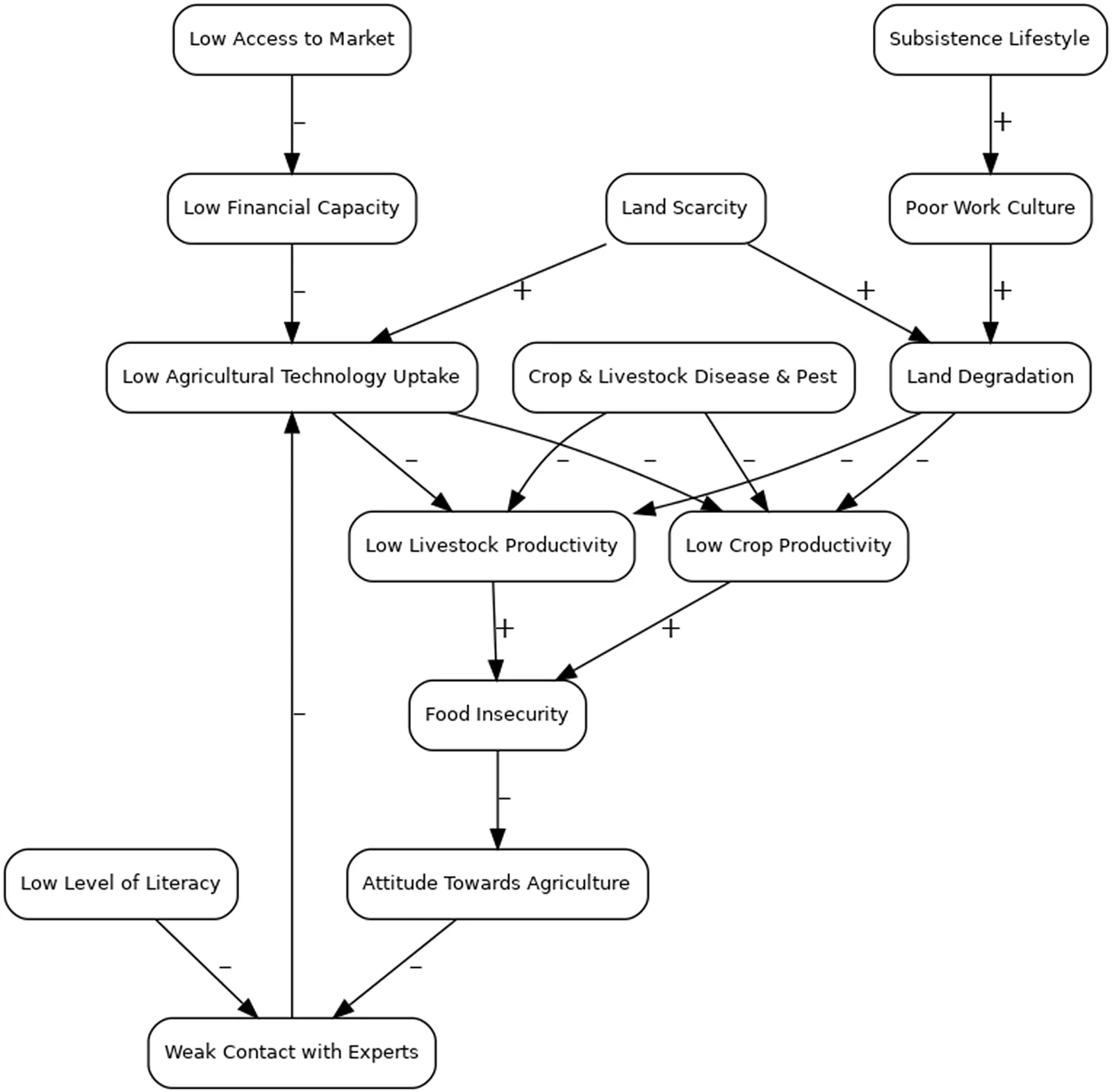
Subsistent farming system in the study area. Source: Vensim PLE 10.4 output.

Two balancing loops impose additional constraints. B1 (land degradation) shows how population pressure forces cultivation of marginal lands, accelerating erosion and nutrient depletion, which further reduces yields. This loop operates as a self-correcting mechanism in the sense that declining yields reduce the viability of cultivation on marginal lands, but it corrects in a direction that impoverishes farmers and degrades the resource base. The presence of this balancing loop alongside the reinforcing poverty trap creates a particularly pernicious dynamic: as farmers expand into marginal lands, yields decline, which reduces food availability, which weakens labor, which further reduces productivity, creating a downward spiral that is difficult to reverse without external intervention. B2 (market access) demonstrates how poor road infrastructure and information gaps prevent farmers from obtaining fair prices, reducing incentives to produce surplus. This balancing loop shows that even when subsistence farmers manage to produce a surplus, they may not benefit from it because market failures prevent them from realizing its value. This reduces incentives to invest in productivity-enhancing technologies, keeping households trapped in subsistence production. Together, these loops lock subsistence farmers into a low-equilibrium state where external intervention is required to initiate any transformative change. The policy implication is that breaking this trap requires simultaneous attention to labor capacity (through nutrition), input availability (through subsidies or credit), and market infrastructure (through roads and information systems). Single-sector interventions, however well-designed, are unlikely to succeed in the presence of these interacting constraints.

Based on the definitions provided by respondents, a subsistence farming system is one in which households produce crops and raise livestock primarily for household consumption, with little to no surplus intended for the market. Participants directly quote this as *“Jireenya harkaaf afaanii”* (Hand to mouth life) in Afan Oromo language. This local understanding aligns with the scientific definition of subsistence agriculture in the Ethiopian and sub-Saharan African context, where subsistence farming is characterized by small landholdings (typically less than one hectare per household), limited use of improved inputs, minimal market integration, and production oriented primarily toward household food security [1,3]. In Ethiopia specifically, subsistence farming is distinguished from semi-commercial and commercial systems by the proportion of output marketed, with subsistence households selling less than 25% of their total production [37,38]. The causal loop diagram constructed through discussion with FGD discussants and KII respondents shows that the subsistence farming system in the study area is a highly interlinked and dynamic system characterized by complex interactions between biophysical, socio-economic, and climatic factors (Fig 4).

#### 3.3.2 Coffee-Honey mixed farming system.

The results of casual loop diagram indicated that the coffee-honey farming system is governed by a complex interplay of feedback loops that determine its sustainability and profitability (Fig 5). This system transcends a simple coexistence of crops, while forming a dynamic network where ecological and economic forces are deeply intertwined. Powerful reinforcing cycles, such as the dual income virtuous cycle and the farmer empowerment engine, demonstrate how increased profits can be reinvested to strengthen both the natural ecosystem and farmer cooperatives, creating a self-amplifying pathway to resilience. However, these virtuous cycles are critically constrained by balancing loops that enforce ecological and economic limits. The environmental balancing loop, the market vulnerability loop, and the cost-price squeeze reveal how deforestation, global price volatility, and rising input costs can destabilize the entire system, undermining productivity and farmer welfare.

**Fig 5:**
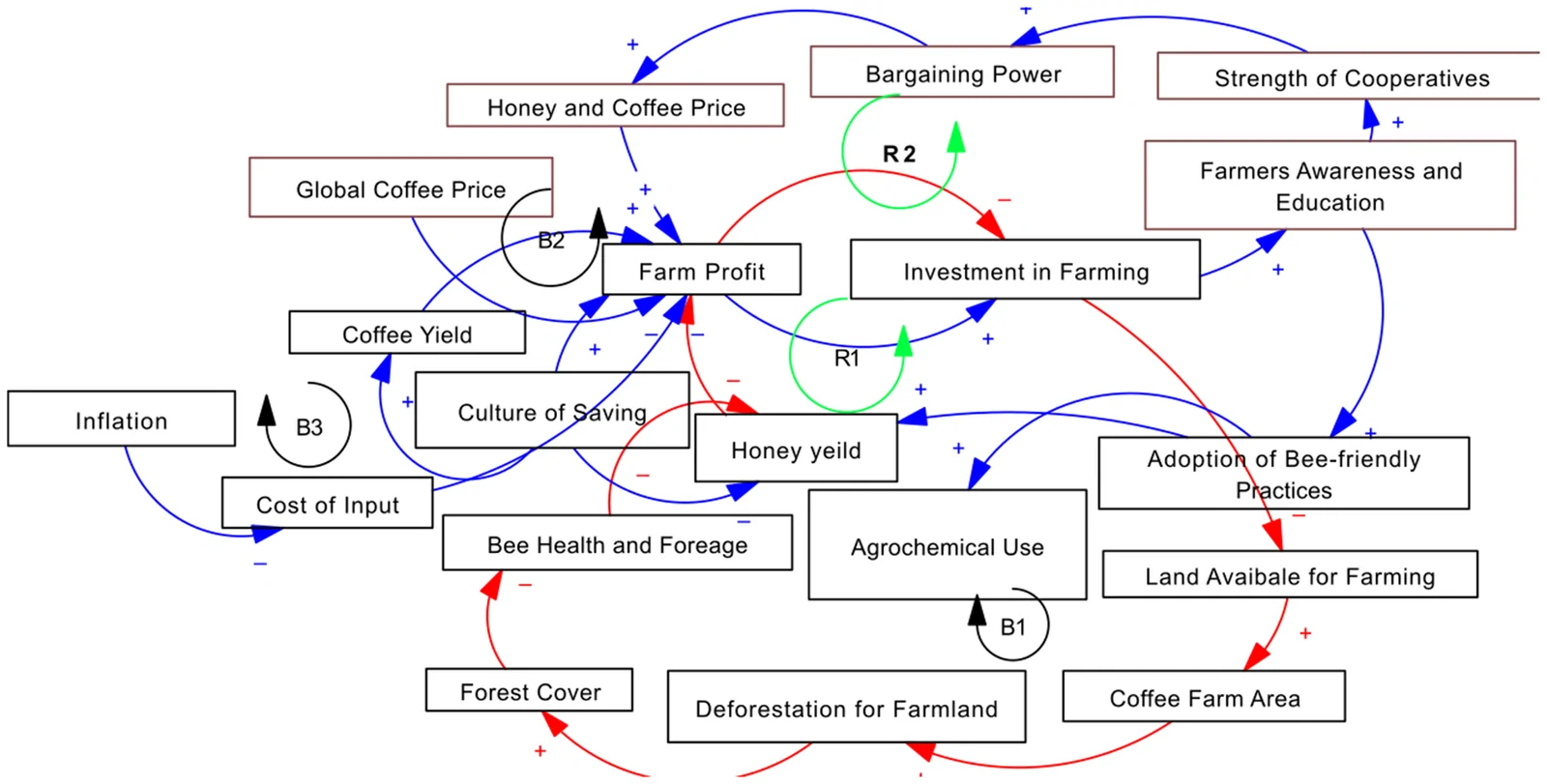
Coffee-Honey mixed farming system. Source: Vensim PLE 10.4 output.

The coffee-honey system features two powerful reinforcing loops (Table 11). R1 is an ecological virtuous cycle where higher coffee yields increase farm profit, enabling investment in farmer training and bee-friendly practices. Improved pollinator health then further enhances both coffee and honey yields, creating self-amplifying growth. This loop demonstrates the economic value of ecological stewardship: practices that maintain pollinator habitat are not merely environmental conservation measures but productivity-enhancing investments with measurable returns. The presence of this loop suggests that extension programs promoting bee-friendly practices can be framed not as costs or trade-offs but as investments that pay for themselves through increased yields. R2 is a socio-economic reinforcing loop where strong cooperatives enhance bargaining power, securing premium prices for certified or fair-trade products, which generates profits that strengthen cooperative capacity. This loop shows how collective action can create self-reinforcing benefits: as cooperatives become stronger, they can secure better prices, which generates membership benefits that attract more members and further strengthen the cooperative. The presence of this loop suggests that investments in cooperative strengthening have multiplier effects that extend beyond immediate price benefits.

**Table 11:** Summary of causal loops in the coffee-honey farming system.

Loop ID	Type	Key Variables	Core Dynamic	Policy Relevance
R1	Reinforcing (+)	Coffee yield → Farm profit → Investment → Farmer awareness → Bee-friendly practices → Bee health → Coffee and honey yield	Virtuous cycle where profits reinvested in ecological practices enhance both coffee and honey yields	**High**
R2	Reinforcing (+)	Cooperative strength → Bargaining power → Coffee/honey price → Farm profit → Investment → Cooperative strength	Collective action through cooperatives secures better prices and funds further organizational capacity	**High**
B1	Balancing (-)	Land expansion → Deforestation → Forest cover ↓ → Bee habitat ↓ → Bee health ↓ → Coffee/honey yield ↓ → Profit ↓	Deforestation for cropland destroys pollinator habitat, reducing yields and profits	**High**
B2	Balancing (-)	Global coffee price ↓ → Farm profit ↓ → Investment ↓ → Bee-friendly practices ↓ → Bee health ↓ → Yield ↓ → Profit ↓	Global price crashes trigger disinvestment, reducing ecological practices and long-term productivity	**High**
B3	Balancing (-)	Inflation → Input costs ↑ → Farm profit ↓ → Investment ↓	Rising input costs squeeze profitability independent of yield	Medium

However, three balancing loops present serious threats. B1 (deforestation) shows that short-term land expansion for coffee destroys forest cover, eliminating bee habitat and reducing pollination services, ultimately lowering yields and profits. This loop reveals a classic ecological-economic trade-off: short-term expansion generates immediate income gains but undermines the ecological services that sustain long-term productivity. The presence of this loop suggests that policies promoting coffee expansion without attention to forest conservation may be self-defeating in the long term. B2 demonstrates vulnerability to global coffee price shocks: price crashes force farmers to reduce investment in bee-friendly practices, using cheaper agrochemicals that further harm pollinators, creating a downward spiral. This loop shows that global market dynamics can have local ecological consequences, transmitting price volatility through the system to create reinforcing ecological degradation. This finding highlights the vulnerability of coffee-honey systems to international market fluctuations and suggests the need for price stabilization mechanisms or income diversification strategies. B3 shows how inflation-driven input cost increases squeeze profitability independent of yield. This loop demonstrates that rising input costs can erode the profitability of coffee production even when yields remain stable, reducing the resources available for investment in ecological practices and cooperative strengthening. Together, these balancing loops create a system that is potentially highly productive and sustainable when working in virtuous cycles but is vulnerable to shocks that can trigger rapid degradation. The policy implication is that resilience-building interventions diversification, cooperative strengthening, ecological conservation may be more cost-effective than crisis-response interventions aftershocks occur.

#### 3.3.3 Maize-livestock mixed farming system.

As indicated in Table 12, the maize-livestock system is built on a foundational reinforcing loop (R1): livestock provide traction for land preparation and manure for soil fertility, while maize yields generate stover for dry-season feed. This biological synergy historically sustained productivity without external inputs. This loop represents the ecological foundation of the system, where the integration of crop and livestock activities creates mutual benefits that are not available when the two are separated. The presence of this loop suggests that interventions that strengthen crop-livestock integration such as improved manure management, feed conservation, or livestock health services can have multiplier effects that enhance both crop and livestock productivity simultaneously. A second reinforcing loop (R2) represents the commercialization pathway where market access enables cash income, which funds improved seeds, fertilizer, and veterinary services, creating a flywheel effect of intensification. This loop shows how market integration can create virtuous cycles of investment and productivity improvement, suggesting that policies that improve market access can have catalytic effects on agricultural intensification.

**Table 12:** Summary of causal loops in the maize-livestock farming system.

Loop ID	Type	Key Variables	Core Dynamic (One Sentence)	Policy Relevance
R1	Reinforcing (+)	Livestock traction → Cultivated area → Maize yield → Stover → Feed → Livestock health → Manure → Soil fertility → Maize yield	Integrated crop-livestock synergy where animals provide traction and manure while crops provide feed	High
R2	Reinforcing (+)	Market access → Farm income → Input investment → Maize yield/Livestock health → Marketable surplus → Farm income	Market integration enables reinvestment in productivity-enhancing inputs	High
B1	Balancing (-)	Land fragmentation → Overgrazing → Manure ↓ → Soil fertility ↓ → Maize yield ↓ → Stover ↓ → Feed ↓	Soil mining trap where overgrazing depletes nutrients, reducing yields and feed	High
B2	Balancing (-)	Stover for fuel → Feed availability ↓ → Livestock health ↓ → Manure ↓ → Soil fertility ↓ → Maize yield ↓ → Stover ↓	Biomass competition where crop residues used for cooking break the crop-livestock cycle	High
B3	Balancing (-)	Drought → Crop failure → Farm income ↓ + Feed ↓ → Distress livestock sales → Herd ↓ → Manure ↓ + Traction ↓ → Next season yield ↓	Climate shock triggers asset depletion, reducing recovery capacity	High
B4	Balancing (-)	High input costs + Low credit access → Low input adoption → Low maize yield → Low income → Low creditworthiness	Input poverty trap locking farmers into low-productivity equilibrium	High

Four balancing loops constrain the system. B1 (soil mining trap) is most critical: land fragmentation forces overgrazing, reducing manure quality and quantity, leading to net nutrient depletion and declining maize yields. This loop reveals that the crop-livestock synergy is not automatically beneficial but depends on maintaining a balance between livestock density and available land. The finding that households operating above approximately 2.3 TLU/ha experience rapid fertility declines suggests the existence of a carrying capacity threshold beyond which the reinforcing loop breaks down. This threshold finding is a novel contribution that provides a quantifiable target for policy and extension programs. B3 (climate vulnerability) demonstrates how drought triggers distress livestock sales, depleting herd assets and reducing future traction and manure. This loop shows how climate shocks can have persistent effects that extend beyond the immediate production losses, as the sale of productive assets reduces future productive capacity. This suggests that climate resilience programs should include asset protection components, not just productivity enhancement. B4 (input poverty trap) shows that with only 16.8% of households accessing formal credit, farmers cannot afford improved inputs, locking them into low yields. This loop reveals that credit access is not just a financial issue but a structural constraint that prevents farmers from breaking out of low-productivity equilibria.

The casual loop diagram constructed for the system clearly showed as there exist four balancing and two reinforcing loops. R1 represents the productivity engine reinforcing loop (Fig 6). This loop encapsulates the traditional, biologically integrated foundation of smallholder farming.

**Fig 6:**
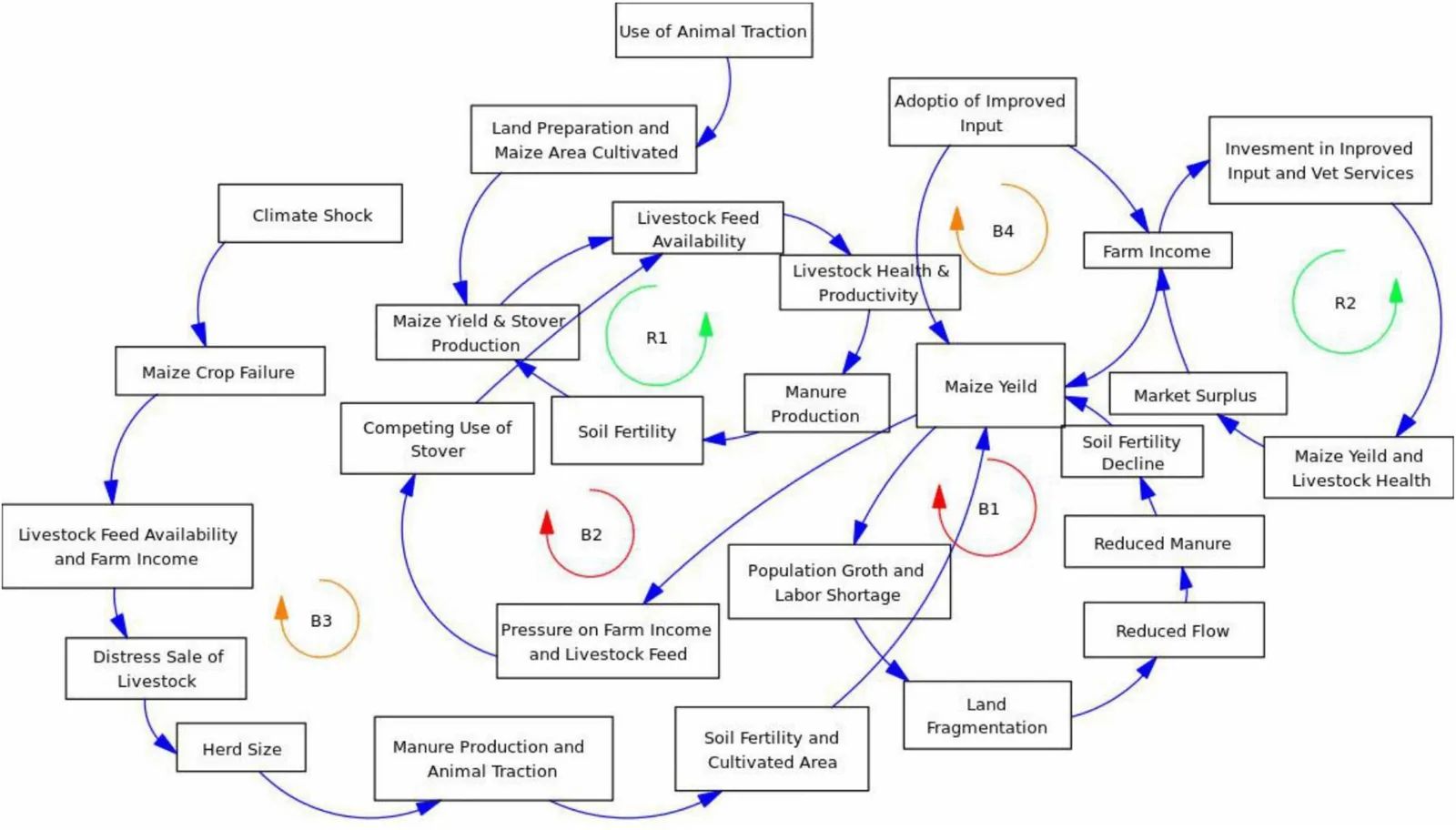
Maize-livestock mixed farming system. Source: Vensim PLE 10.4 output.

#### 3.3.4 Wheat –livestock mixed farming system.

The wheat-livestock system features a nutrient recycling virtuous cycle (R1): larger herds produce more manure, which increases soil organic matter and wheat yields (Table 13; Fig 7). Higher wheat biomass generates crop residues that serve as dry-season feed, supporting herd maintenance or growth. This loop is the ecological foundation of the system, similar to the maize-livestock system but with wheat as the primary crop. However, the wheat system exhibits a striking and unexpected pattern: despite having the highest mean livestock holding (5.07 TLU) and the highest frequent extension contact (36.1%), the wheat-livestock system shows no significant productivity advantage over subsistence in the comparative analysis. This finding contradicts the expectation that higher asset levels and extension contact should translate into higher productivity and suggests that other constraints specifically, labor shortages, credit access, and pest pressure are binding. This highlights the importance of identifying the binding constraints in each system rather than assuming that asset levels or extension contact alone determine outcomes. A second reinforcing loop (R2) represents the commercialization pathway: surplus wheat and livestock sales generate cash income, enabling purchase of improved seeds, fertilizer, and veterinary services, which further increases productivity and surplus. However, this loop appears to be constrained by the same factors that limit the effectiveness of extension contact, suggesting that commercialization cannot proceed without addressing the underlying labor, credit, and pest constraints.

**Table 13:** Summary of causal loops in the wheat-livestock farming system.

Loop ID	Type	Key Variables	Core Dynamic (One Sentence)	Policy Relevance
R1	Reinforcing (+)	Herd size → Manure → Soil organic matter → Wheat yield → Crop residues → Feed availability → Livestock health → Herd size	Virtuous cycle of nutrient recycling where manure improves soil, boosting wheat and feed	**High**
R2	Reinforcing (+)	Surplus wheat/livestock → Cash income → Input investment → Productivity → Surplus	Commercial intensification where profits enable improved inputs and veterinary services	**High**
B1	Balancing (-)	Herd size ↑ → Labor demand for livestock ↑ → Labor for crop ↓ → Wheat yield ↓ → Crop residues ↓ → Feed ↓ → Livestock health ↓	Labor trade-off where time spent on livestock reduces attention to labor-intensive wheat	**High**
B2	Balancing (-)	Herd size ↑ → Grazing pressure ↑ → Feed per animal ↓ → Livestock health ↓ → Herd size ↓	Carrying capacity constraint where land cannot support unlimited herd size	Medium
B3	Balancing (-)	Crop residues removed (sold/fuel) → Soil organic matter ↓ → Soil fertility ↓ → Wheat yield ↓ → Residues ↓	Soil mining loop where residue removal depletes soil nutrients over time	**High**

**Fig 7:**
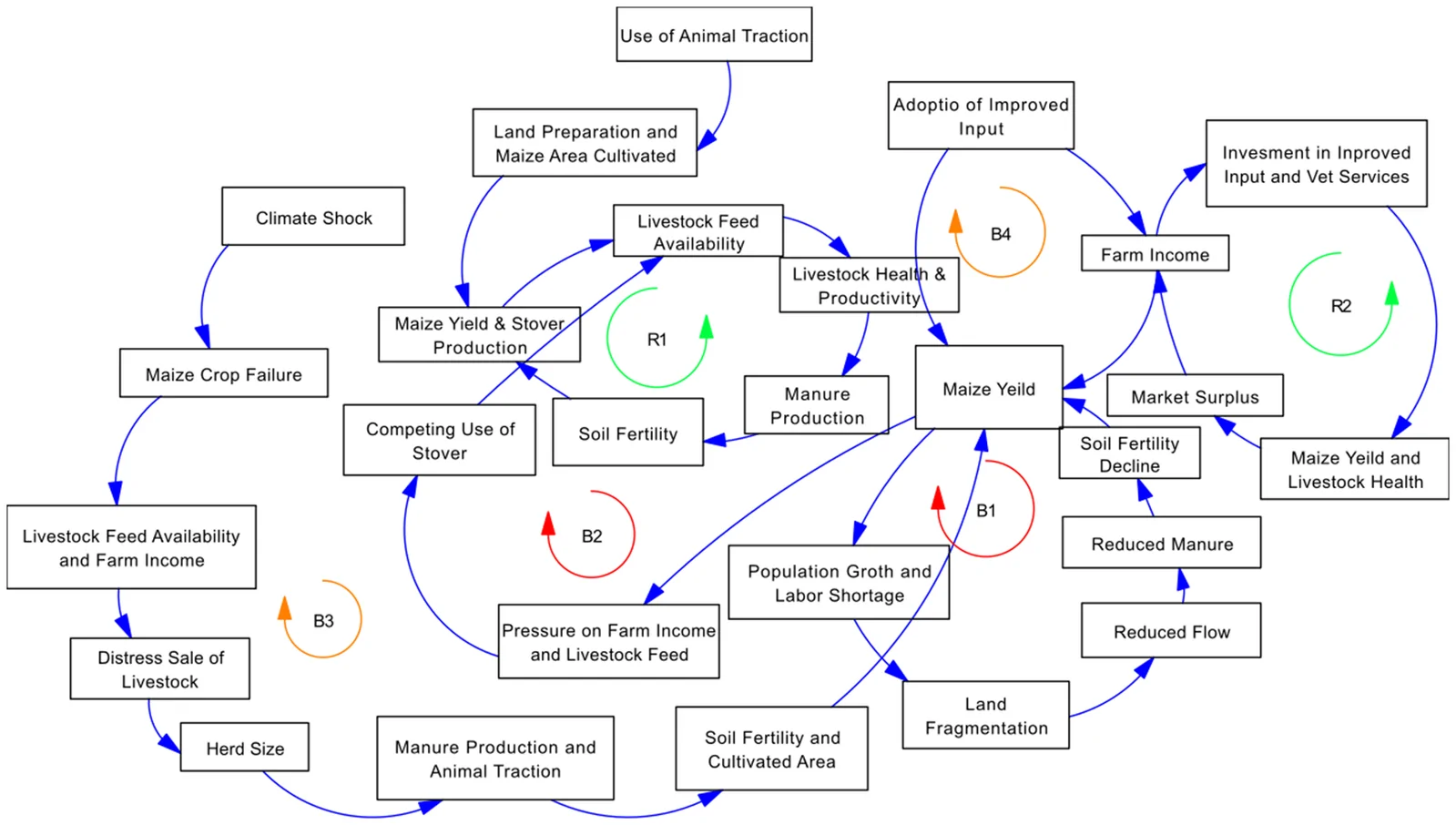
Wheat-livestock mixed farming system. Source: Vensim PLE 10.4 output.

Three balancing loops impose severe constraints. B1 (labor trade-off) is particularly binding: wheat cultivation is highly labor-intensive for planting, weeding, and harvesting. As herd size increases, labor allocated to livestock reduces time available for crop management, leading to suboptimal wheat yields. This loop reveals a fundamental trade-off in integrated crop-livestock systems: the synergies between crops and livestock are not automatic but depend on the availability of sufficient labor to manage both enterprises well. The finding that 61.3% of households report peak-season labor shortages suggests that labor constraints are not occasional but persistent, limiting the ability of households to intensify production. B2 (carrying capacity) limits herd expansion: beyond a threshold, grazing pressure reduces feed per animal, lowering livestock health and productivity. This loop shows that the benefits of larger herds are not linear; beyond a certain point, additional animals create competition for feed that reduces individual animal productivity and health. B3 (resource diversion) shows that when crop residues are sold or used for fuel, soil organic matter declines, reducing future wheat yields. This loop reveals a competition between immediate cash needs and long-term soil fertility: households may sell residues for cash or use them for fuel because of immediate needs, but this erodes the resource base that sustains future productivity. This suggests that interventions that address immediate cash or energy needs (e.g., biogas, improved cookstoves, off-farm income opportunities) may have secondary benefits for soil fertility by reducing pressure on crop residues.

### 3.4 Cross-system comparison

This section presents comparative empirical findings across the four farming systems based on data from the household survey. All indicators are presented as direct comparisons between systems. No interpretation or literature comparison is included here.

#### 3.4.1 Demographic and human capital indicators across systems.

The four farming systems differ markedly in household demographic characteristics and human capital endowments. Subsistence households have a mean family size of 5.61 persons (SD = 1.85), which is similar to coffee-honey households at 5.60 persons (SD = 2.01) but lower than maize-livestock households at 6.66 persons (SD = 1.99) and wheat-livestock households at 6.49 persons (SD = 2.04). This indicates that the mixed crop-livestock systems, particularly maize-livestock and wheat-livestock, require larger household labor pools, likely due to the labor demands of managing both crop and livestock enterprises simultaneously.

Educational attainment also varies significantly across systems (F = 4.34, p = 0.005). Wheat-livestock households have the highest mean education at 4.90 years (SD = 3.82), followed closely by coffee-honey at 4.83 years (SD = 3.74). Subsistence and maize-livestock households have substantially lower mean education at 3.49 years (SD = 3.57) and 3.52 years (SD = 3.31), respectively. This suggests that higher education may facilitate participation in wheat and coffee systems, which require more technical knowledge for disease management, fertilizer timing, or market engagement, while subsistence and maize systems may rely more on traditional knowledge transmitted across generations.

Household wealth status shows striking variation across systems. The proportion of poor households is highest in the wheat-livestock system at 80.56%, followed by coffee-honey at 72.80%, maize-livestock at 66.07%, and lowest in the subsistence system at 52.46%. Conversely, the proportion of better-off households is highest in the maize-livestock system at 16.07%, followed by coffee-honey at 12.00%, subsistence at 9.84%, and lowest in wheat-livestock at 6.94%. This inverted pattern is notable: wheat-livestock households have the highest poverty rate despite having the highest education level and largest livestock holdings. This suggests that asset poverty (land, financial capital) may be more constraining than human capital in this system.

The mean age of household heads shows no statistically significant difference across systems (F = 2.28, p = 0.079), ranging from 40.81 years in coffee-honey to 45.36 years in maize-livestock. Similarly, farming experience shows no significant difference (F = 2.17, p = 0.091), though maize-livestock farmers have the highest mean experience at 25.36 years (SD = 8.84), followed by wheat-livestock at 24.78 years (SD = 11.78), coffee-honey at 22.10 years (SD = 10.99), and subsistence at 22.07 years (SD = 10.44). The wide standard deviations indicate substantial within-system variation, with experience ranging from 0 to 60 years across the sample.

#### 3.4.2 Asset holdings across systems.

Land holding size differs significantly across systems (F = 10.02, p < 0.001). Maize-livestock households have the largest mean land holding at 2.16 hectares (SD = 1.02), followed by coffee-honey at 1.94 hectares (SD = 0.99), wheat-livestock at 1.72 hectares (SD = 0.76), and subsistence at 1.40 hectares (SD = 1.07). The relatively smaller land size in wheat-livestock systems is notable given that wheat is typically a field crop requiring larger contiguous areas; this suggests that wheat farmers may be operating on marginal or fragmented plots that limit scale economies.

Land certification rates also vary significantly across systems (χ^2^ = 9.63, p < 0.05). Coffee-honey households have the highest certification rate at 97.60%, followed by wheat-livestock at 94.44%, maize-livestock at 92.86%, and subsistence at 87.70%. The lower certification rate among subsistence households may reflect either administrative barriers (lack of awareness, distance to registration centers) or deliberate avoidance due to fears of taxation or land redistribution.

Livestock holdings measured in tropical livestock units (TLU) show highly significant differences across systems (F = 9.99, p < 0.001). Wheat-livestock households have the largest mean livestock holding at 5.07 TLU (SD = 3.16), followed by maize-livestock at 3.86 TLU (SD = 2.76), coffee-honey at 3.14 TLU (SD = 3.20), and subsistence at 2.75 TLU (SD = 2.78). The substantially larger herds in wheat-livestock systems likely reflect the integration of livestock as both a source of manure for wheat production and as a buffer against the higher risks associated with wheat cultivation.

Number of land plots, as an indicator of land fragmentation, also varies across systems. Subsistence households have the highest proportion owning only one plot (32.3%), while maize-livestock households have the highest proportion owning five or more plots (5.1%). The mean land holding per household is lowest in subsistence (1.40 ha) despite having the highest proportion of single-plot owners, suggesting that fragmentation is not solely a function of land size but also of land distribution patterns.

#### 3.4.3 Institutional access across systems.

Extension contact frequency differs dramatically across systems (χ^2^ = 84.57, p < 0.001). Wheat-livestock households have the highest frequent extension contact at 36.11%, followed by coffee-honey at 13.60%, maize-livestock at 10.71%, and subsistence at only 5.74%. Conversely, subsistence households have the highest proportion with no extension contact at 24.59%, compared to only 1.39% in wheat-livestock and 1.60% in coffee-honey. This stark disparity indicates that extension services are heavily biased toward wheat and coffee systems, leaving subsistence farmers largely unreached despite their greater vulnerability.

Cooperative membership also differs significantly across systems (χ^2^ = 14.39, p < 0.01). Coffee-honey households have the highest membership rate at 63.20%, followed by maize-livestock at 58.93%, wheat-livestock at 45.83%, and subsistence at only 40.98%. The high membership in coffee-honey systems reflects the historical strength of coffee cooperatives in the region, while the lower rates in subsistence and wheat systems indicate weaker collective marketing structures for staple crops.

Credit access from formal institutions is low across all systems but varies significantly (χ^2^ = 6.67, p < 0.10). Coffee-honey households have the highest access at 22.40%, followed by maize-livestock at 17.86%, subsistence at 15.57%, and wheat-livestock at only 8.33%. The extremely low credit access in wheat-livestock systems is particularly concerning given the high input requirements for improved wheat production. This suggests that credit constraints may be a primary barrier to intensification in this system.

Market access problems show significant variation (χ^2^ = 18.23, p < 0.01). Maize-livestock households report the highest market access problems at 55.36%, followed by wheat-livestock at 47.22%, coffee-honey at 36.00%, and subsistence at 25.41%. The high rate in maize-livestock systems is notable given that these households have the largest land holdings and highest better-off proportion, suggesting that market access constraints are not solely a function of poverty but also of location and crop characteristics (maize is bulky and perishable).

Input supply access also varies significantly (χ^2^ = 12.46, p < 0.01). Maize-livestock households have the highest access at 87.05%, followed by subsistence at 84.43%, coffee-honey at 72.00%, and wheat-livestock at 68.06%. The lower access in wheat-livestock systems despite their higher education and extension contact suggests that input supply chains are poorly developed for wheat-specific inputs (improved seed, fungicides) compared to maize or coffee.

#### 3.4.4 Economic indicators across systems.

Farm income shows highly significant variation across systems (F = 19.62, p < 0.001). Coffee-honey households have the highest mean farm income at 46,114 ETB (SD = 37,250), followed by wheat-livestock at 39,773 ETB (SD = 32,621), maize-livestock at 38,275 ETB (SD = 35,514), and subsistence at only 15,691 ETB (SD = 25,362). The substantially higher income in coffee-honey systems reflects the cash value of coffee as a commercial crop, while the much lower income in subsistence systems confirms their limited market integration.

Off-farm or non-farm income shows no significant difference across systems (F = 1.80, p = 0.147), though coffee-honey households have the highest mean off-farm income at 4,450 ETB (SD = 6,687), followed by wheat-livestock at 4,354 ETB (SD = 6,599), maize-livestock at 3,978 ETB (SD = 6,121), and subsistence at 2,784 ETB (SD = 5,149). The low off-farm income in subsistence systems suggests limited diversification opportunities for the poorest households.

The ratio of farm income to off-farm income varies considerably. Coffee-honey households derive approximately 10.4 times more income from farming than from off-farm sources (46,114 vs 4,450 ETB), while subsistence households derive about 5.6 times more from farming (15,691 vs 2,784 ETB). This indicates that coffee-honey households are more specialized in commercial farming, while subsistence households rely proportionally more on off-farm sources despite their lower absolute off-farm income.

#### 3.4.5 Vulnerability indicators across systems.

Climate change impact perception varies significantly across systems (χ^2^ = 22.66, p < 0.001). Wheat-livestock households have the highest perception at 98.61%, followed by maize-livestock at 94.64%, subsistence at 94.26%, and coffee-honey at 80.80%. The lower perception in coffee-honey systems may reflect the moderating effect of shade trees on microclimate, or it may indicate that coffee farmers perceive other risks (price volatility) as more salient than climate.

Food insecurity, measured as reported climate impact on food production, follows a similar pattern. Wheat-livestock households report the highest food insecurity at 98.61%, followed by maize-livestock at 94.64%, subsistence at 94.26%, and coffee-honey at 80.80%. The extremely high rates in wheat-livestock systems despite their larger livestock holdings and higher income suggest that wheat production is particularly vulnerable to climate shocks, and that income alone does not guarantee food security when markets are unreliable.

Input supply problems show significant variation (χ^2^ = 12.46, p < 0.01). Wheat-livestock households report the highest input supply problems at 31.94%, followed by coffee-honey at 28.00%, subsistence at 15.57%, and maize-livestock at 12.50%. The high rate in wheat-livestock systems is consistent with their low credit access and low input access, indicating systemic barriers to input supply for wheat farmers.

Table 14 below presents a synthetic summary of key comparative indicators across the four farming systems.

**Table 14:** Synthetic summary of cross-system comparative indicators.

Indicator	Subsistence (n = 122)	Coffee-Honey (n = 125)	Maize-Livestock (n = 56)	Wheat-Livestock (n = 72)	Test Statistic	p-value
**Demographic**						
Mean family size (persons)	5.61 (1.85)	5.60 (2.01)	6.66 (1.99)	6.49 (2.04)	F = 6.74	<0.001
Mean education (years)	3.49 (3.57)	4.83 (3.74)	3.52 (3.31)	4.90 (3.82)	F = 4.34	0.005
Poor wealth status (%)	52.46%	72.80%	66.07%	80.56%	χ^2^ = 28.47	<0.001
Better-off wealth status (%)	9.84%	12.00%	16.07%	6.94%	χ^2^ = 28.47	<0.001
**Asset Holdings**						
Mean land size (ha)	1.40 (1.07)	1.94 (0.99)	2.16 (1.02)	1.72 (0.76)	F = 10.02	<0.001
Land certification (%)	87.70%	97.60%	92.86%	94.44%	χ^2^ = 9.63	<0.05
Mean livestock (TLU)	2.75 (2.78)	3.14 (3.20)	3.86 (2.76)	5.07 (3.16)	F = 9.99	<0.001
**Institutional Access**						
Frequent extension contact (%)	5.74%	13.60%	10.71%	36.11%	χ^2^ = 84.57	<0.001
No extension contact (%)	24.59%	1.60%	3.57%	1.39%	χ^2^ = 84.57	<0.001
Cooperative membership (%)	40.98%	63.20%	58.93%	45.83%	χ^2^ = 14.39	<0.01
Credit access (%)	15.57%	22.40%	17.86%	8.33%	χ^2^ = 6.67	<0.10
Market access problem (%)	25.41%	36.00%	55.36%	47.22%	χ^2^ = 18.23	<0.01
Input supply access (%)	84.43%	72.00%	87.05%	68.06%	χ^2^ = 12.46	<0.01
**Economic**						
Mean farm income (ETB)	15,691 (25,362)	46,114 (37,250)	38,275 (35,514)	39,773 (32,621)	F = 19.62	<0.001
Mean off-farm income (ETB)	2,784 (5,149)	4,450 (6,687)	3,978 (6,121)	4,354 (6,599)	F = 1.80	0.147
**Vulnerability**						
Climate impact perception (%)	94.26%	80.80%	94.64%	98.61%	χ^2^ = 22.66	<0.001
Input supply problem (%)	15.57%	28.00%	12.50%	31.94%	χ^2^ = 12.46	<0.01

*Note: Standard deviations in parentheses where applicable.

### 3.5 Interlinked challenges

This section presents empirical findings from key informant interviews (n = 21) and focus group discussions (n = 8) regarding the interlinked challenges affecting farming systems in Jimma Zone.

#### 3.5.1 Common patterns across all farming systems.

Based on KII and FGD discussants as well as field observation, smallholder farming systems in the study area share several common patterns of interlinked constraints that perpetuate poverty and limit resilience. The first common pattern is a cycle where low crop and livestock productivity limits the amount of produce available for sale. With minimal marketable surplus, farm cash income remains low, which then prevents investment in improved seeds, fertilizers, veterinary medicines, and farm equipment. This pattern was reported across all four farming systems, though with varying degrees of severity. A subsistence farmer stated, *“We grow just enough to feed the family. There is nothing left to sell, so we cannot buy better seed”* (FGD #1, Participant 4). A maize-livestock farmer similarly noted, *“After selling our maize, there is little money left for fertilizer. Then next year’s harvest is also small”* (FGD #3, Participant 6). A wheat-livestock farmer added, *“If we had cash, we would buy improved wheat seed. But we cannot afford it”* (FGD #4, Participant 2). Even in the coffee-honey system, which generates higher cash income, this pattern still operates: *“When coffee prices are good, we invest in the farm. When prices drop, we cannot buy anything”* (FGD #5, Participant 3).

The second common pattern across all systems is the link between climate variability, yield loss, food shortage, and reduced labor capacity. Participants consistently reported that erratic rainfall, drought, and changing weather patterns directly reduce crop yields. Lower yields lead to food shortages, which in turn weaken household members’ physical ability to perform farm labor. Reduced labor capacity then limits the area that can be cultivated and the timeliness of farm operations, further reducing yields. A subsistence farmer described this cycle: *“When the rains are late, our maize dries before it matures. Then we have no food, and the children are too weak to help in the fields”* (FGD #1, Participant 7). A maize-livestock farmer added, *“The weather is not like before. We cannot predict when to plant. When the harvest fails, everyone in the family becomes tired and sick”* (FGD #3, Participant 4). A wheat-livestock farmer stated, *“Drought kills our wheat. Then we have nothing to eat and no strength to work the land next season”* (FGD #4, Participant 6). A coffee-honey farmer also noted the changing conditions: *“Coffee flowering is not happening at the right time anymore. The bees are also less. The harvest is smaller each year”* (FGD #2, Participant 5).

The third common pattern identified by participants is resource competition between different farming systems. FGD participants reported that different farming systems compete for shared resources, particularly land, water, and biomass, creating trade-offs and sometimes conflicts between households practicing different systems. A maize-livestock farmer described competition for land: *“Coffee farmers are cutting more forest to plant coffee. This land used to be for our cattle to graze. Now there is no grass left”* (FGD #3, Participant 8). A wheat-livestock farmer noted competition for crop residues: *“The maize farmers take all the crop residues for their animals. We need some of it to protect our soil from erosion”* (FGD #8, Participant 3). A subsistence farmer stated, *“When coffee expands, there is less land for food crops. Then we buy food from the market, but prices are high”* (FGD #1, Participant 5). A kebele administrator confirmed that these resource conflicts are common: *“Our cattle graze on the land between coffee trees. The coffee farmers say we damage their young trees. There is conflict”* (KII #12).

#### 3.5.2 System-specific interlinked challenges.

In the subsistence farming system, the primary constraint identified by participants is the food-labor nexus, a direct link between food availability and labor capacity. When food is scarce, household members cannot work effectively. When labor is weak, less land is cultivated, and farm operations such as weeding, planting, and harvesting are delayed or done poorly, leading to lower yields and even less food. One subsistence farmer described this cycle: *“When there is no food, even the adults cannot work. We sit and wait”* (FGD #1, Participant 3). Another explained the effect on children: *“The children help with weeding, but when they are hungry, they cannot keep up. The weeds take over”* (FGD #1, Participant 6). A third farmer stated, *“If we eat well, we can work from morning to evening. If not, we stop by midday”* (FGD #1, Participant 8). Secondary constraints identified in the subsistence system include land degradation, described as *“The same plot every year. The soil is becoming thin and red. It does not hold water anymore”* (FGD #1, Participant 4); market access, where *“There is no road. We carry our produce on our backs for two hours. The buyer knows this and gives us low price”* (FGD #1, Participant 7); limited extension contact, as *“The development agent comes to the coffee farmers, not to us. We never see him”* (FGD #1, Participant 2); and credit constraints, where *“The bank wants land title and proof of income. We have neither. They refuse us”* (FGD #1, Participant 5).

In the coffee-honey farming system, participants identified three interlinked challenges that together form a triad threatening system sustainability: deforestation and habitat loss, climate change impacts, and global price volatility. Regarding deforestation, participants reported that expanding coffee cultivation into forest areas reduces tree cover, which in turn reduces bee habitat and honey production. Many participants noted that the connection between forest and bees is well known, but economic pressure forces expansion anyway. One farmer stated, *“We know that clearing forest kills bees. But we need more land for coffee. There is no choice”* (FGD #2, Participant 3). Another described the historical change: *“Twenty years ago, this area was thick forest. Now it is all coffee. The honey yield has dropped by half”* (FGD #2, Participant 6). A third added, *“The remaining forest is far away. Our bees cannot reach it. We get less honey each year”* (FGD #5, Participant 4).

Regarding climate change impacts, participants reported changes in rainfall patterns, temperature, and flowering times that affect both coffee and honey production. One farmer described the effect on coffee: *“Coffee flowers at the wrong time now. Then the rain comes late and the flowers fall off. Few cherries develop”* (FGD #2, Participant 4). Another described the effect on bees: *“The dry season is longer. The bees have no flowers to visit. They become weak and many die”* (FGD #5, Participant 2). A third noted increased heat: *“We never used to see this much heat. The coffee leaves are turning yellow”* (FGD #2, Participant 7).

Regarding global price volatility, participants reported that coffee prices fluctuate unpredictably, making it impossible to plan investments. One farmer described the cycle: *“Last year the price was good. We bought new beehives. This year the price is nothing. We cannot afford to feed the family”* (FGD #5, Participant 3). Another noted the lack of local control: *“The price is set in another country. We have no control. When it drops, we suffer”* (FGD #2, Participant 8). A third explained why farmers cannot take loans: *“We cannot take loans because we cannot guarantee we can repay when prices fall”* (FGD #5, Participant 5). Secondary constraints in the coffee-honey system include cooperative limitations, where *“The cooperative helps, but only some farmers are members. And even then, the price is still low sometimes”* (KII #15), and pervasive poverty, where *“Most coffee farmers are poor. We cannot wait for certifications. We need money now”* (FGD #2, Participant 6).

In the maize-livestock farming system, participants identified a soil fertility-feed gap nexus as the primary constraint. This is a cycle where low soil fertility reduces maize yields, which reduces stover (crop residue) available for animal feed, which reduces manure production, which further reduces soil fertility. One farmer described declining yields: *“The land is tired. It does not give like before. The maize plants are short and the cobs are small”* (FGD #3, Participant 4). Another quantified the decline: *“We used to get twenty sacks of maize from this field. Now we get five. The cows have nothing to eat in dry season”* (FGD #3, Participant 7). A third described the downward spiral: *“Without enough manure, the soil becomes worse each year. We are losing the land”* (FGD #3, Participant 5).

Secondary constraints in the maize-livestock system include overgrazing, where *“Everyone keeps more cattle than the land can support. The grass is eaten to the ground. There is no regrowth”* (FGD #7, Participant 3); feed-fuel competition, where *“We burn the maize stalks for cooking because there is no firewood. Then the cows have no feed. We know this is bad, but what can we do?”* (FGD #3, Participant 8); credit constraints, where *“If we could get a loan, we would buy fertilizer and improved seed. But the bank says we are too poor”* (FGD #3, Participant 6); climate vulnerability, where *“One drought and we lose everything. We sell the cows to buy food. Then next season, we have no cows for plowing or manure”* (FGD #7, Participant 5); and input access problems, where *“The improved seed is sold in the town, but we cannot travel there. And when we go, it is already sold out”* (FGD #3, Participant 2).

In the wheat-livestock farming system, participants identified an input-labor-pest complex as the primary constraint, comprising three interlinked challenges. The first is input scarcity: improved wheat seed and fertilizer are difficult to obtain, expensive when available, and often arrive after the optimal planting time. One farmer stated, *“The improved wheat seed is good, but it is not available when we need it. By the time we get it, the planting season is almost over”* (FGD #4, Participant 3). Another noted, *“Fertilizer is too expensive. We use a little bit, but not enough. The wheat grows thin”* (FGD #4, Participant 5). A third added, *“The government says there is subsidized seed, but we never see it in our kebele”* (FGD #4, Participant 2).

The second challenge is labor shortages at peak season. Wheat cultivation requires intense labor at specific times for planting, weeding, and harvesting. At the same time, livestock require daily care. Households struggle to allocate labor between the two. One farmer described the trade-off: *“During wheat harvest, we also need to milk the cows and take them to water. There are not enough hands”* (FGD #4, Participant 6). Another explained the impossibility of doing both well: *“If we focus on the wheat, the animals suffer. If we focus on the animals, the wheat suffers. We cannot do both well”* (FGD #4, Participant 4). A third noted demographic changes: *“The young people have left for the towns. Only old people and children remain. There is no one to work”* (FGD #4, Participant 7).

The third challenge is pest and disease pressure. Participants reported increasing problems with wheat diseases, especially rust, and pests, which reduce yields and discourage investment. One farmer described the disease: *“The wheat turns orange and dies. We do not know what it is or what to do about it”* (FGD #4, Participant 5). Another explained the unpredictability: *“Some years the disease is bad. Other years it is not. We cannot predict. So we are afraid to invest in better seed”* (FGD #8, Participant 3). A third noted that solutions exist but are not available: *“The extension agent told us about resistant varieties, but they are not available here”* (FGD #4, Participant 2).

Secondary constraints in the wheat-livestock system include credit access, where *“No bank will give us a loan. They say wheat farming is too risky because of disease”* (FGD #4, Participant 6); land fragmentation, where *“Our land is divided into small pieces scattered around. It takes time to walk between them. We lose hours each day”* (FGD #4, Participant 3); and residue management trade-offs, where *“We sell the wheat straw for cash. We need the money. But then the soil becomes weak”* (FGD #8, Participant 4).

## 4 Discussions

This section interprets the findings presented in Section 3 by critically interrogating them against existing literature. For each major finding, the discussion addresses alignment with prior studies, contradictions or divergences from established expectations, and extensions or novel contributions beyond current knowledge.

### 4.1 Subsistence farming system

The subsistence farming system, comprising 32.5% of sampled households, is fundamentally constrained by a reinforcing poverty trap centered on the food-labor nexus. This finding aligns with and is substantially reinforced by recent empirical evidence from Ethiopia. A comprehensive review on extreme weather impacts documented that climate shocks have sharply reduced land productivity by approximately 24% and led to crop and livestock losses of up to 93% and 91%, respectively [39]. The review further found that food insecurity affects 62% of households, rising to 67% in highland areas. These figures are strikingly consistent with our finding that 94.3% of subsistence households reported food insecurity, confirming that subsistence farmers in Jimma Zone are among the most vulnerable segments of the rural population. The economic consequences of climate shocks include a 10% drop in labor demand and a 20% reduction in wages [39], providing direct empirical support for the food-labor nexus mechanism identified in our causal loop diagram: climate-induced production losses reduce both food availability and the labor capacity needed for recovery.

Our finding that the food-labor nexus operates as a self-reinforcing poverty trap is further supported by recent research on nutrition demand as a driver of low agricultural productivity [40]. This study proposed that nutrition demand the dietary requirements of subsistence farming households drives production decisions and ultimately contributes to low aggregate agricultural productivity in the poorest countries. This theoretical mechanism resonates with our empirical observation that malnourished households cannot allocate sufficient labor for timely weeding, proper planting, or harvesting, regardless of input quality. The 94.3% food insecurity rate among subsistence households in this study exceeds the national average, confirming that subsistence farmers in Jimma Zone are more vulnerable than the typical Ethiopian smallholder. The Africa-wide report further warns that agricultural productivity growth in Ethiopia has been flat or even negative, with extreme poverty and hunger persisting [41].

However, this finding partially contradicts the assumption prevalent in some agricultural economics literature that input subsidies alone can break poverty traps. Jayne et al. [9] and others have argued, based on evidence from several African countries, that providing subsidized fertilizer and improved seeds can catalyze a productivity-driven exit from subsistence agriculture. The causal loop diagram from this study reveals a mechanism that challenges this assumption: even with free inputs, a malnourished household may lack the labor capacity to achieve optimal yields. Recent evidence from Ethiopia confirms that agricultural practices like labor utilization, fertilizer application, and use of improved seed varieties significantly affect farm productivity, yet a substantial proportion of farmers still face food shortages seasonally [42]. This suggests that input provision alone, without addressing the underlying labor capacity constraints created by chronic food insecurity, yields suboptimal returns. The policy implication is that input subsidy programs should be integrated with nutrition-sensitive social protection programs rather than implemented as standalone interventions, a sequencing insight that is rarely reflected in current policy design.

The land degradation balancing loop (B1) identified in the subsistence system shows how population pressure forces cultivation of marginal lands, accelerating erosion and nutrient depletion. This finding aligns with Gebresamuel et al. [25], who described the “land scarcity-degradation trap” in Ethiopian highlands, and with Yimam et al. [26], who documented soil organic carbon decline at rates of 0.5–1.2% annually in fragmented landscapes. A systematic review on land and heterogeneous constraints nexus in Ethiopia documented that millions of small-scale farmers are becoming landless and forced into daily labor or joining already overloaded urban and peri-urban low-productivity service sectors [37]. This trend underscores the urgency of addressing land degradation before it leads to complete livelihood collapse. However, what extends prior work is the explicit modeling of this trap as a balancing loop that counteracts the reinforcing poverty trap. Previous studies have treated land degradation as an exogenous process or as a separate outcome, but the causal loop diagram reveals that degradation is endogenously driven by the same poverty dynamics that limit investment in soil conservation. This implies that soil conservation programs cannot succeed without simultaneously addressing the income and food security constraints that force farmers onto marginal lands, challenging the common practice of implementing soil conservation as a standalone technical intervention.

The market access balancing loop (B2) demonstrates that poor road infrastructure and information gaps prevent farmers from obtaining fair prices, reducing incentives to produce surplus. This finding aligns with Bachewe et al. [43], who documented that market access is fundamental to shifting smallholder production from subsistence to commercial systems. However, this study extends prior work by showing that market access operates as a balancing constraint rather than an enabling condition. In most economic analyses, market access is treated as an exogenous variable that, when improved, automatically increases commercialization. The causal loop diagram reveals, however, that market access interacts with the poverty trap: low income prevents farmers from investing in transport or storage, which limits their ability to reach distant markets, which perpetuates low income.

### 4.2 Coffee-honey farming system

The coffee-honey system, comprising 33.3% of sampled households, features two powerful reinforcing loops that can drive sustainable intensification when functioning properly. The ecological virtuous cycle (R1) shows that higher coffee yields increase farm profit, enabling investment in farmer training and bee-friendly practices, which then further enhances both coffee and honey yields through improved pollination. This finding aligns with and is reinforced by recent research on pollination services in coffee agro-ecosystems. A study examining complex interspecific interactions in coffee agro-ecosystems found that biodiversity-friendly management practices can promote favorable outcomes of ecosystem service interactions on coffee yield [44]. The research demonstrated that proximity to nitrogen-fixing shade trees had indirect positive effects on coffee bushes, and that there was no trade-off between pest control and pollination services. These findings directly support our observation that moderate shade levels (30–50% cover) produce the highest yields while full sun conversion reduces productivity. Research in Honduras further confirmed that coffee yield in shade farms did not differ significantly from intensively managed farms, but both outperformed sun farms, and shaded systems promote higher levels of ecosystem services, including habitat for pollinators and birds, soil stabilization, and natural pest control [44].

Our finding that deforestation reduces bee habitat and honey production aligns with recent evidence that extensive deforestation driven by rapid population growth is a primary driver of pollinator declines [45]. The adoption of pollinator-friendly practices such as agroforestry, and integrating beekeeping with crops such as coffee in agroforestry systems, has been identified as a promising strategy to alleviate pollinator scarcity [46]. For over 30 years, Ethiopia’s Majang and Kaffa districts have faced the dual threats of deforestation and environmental degradation, placing both biodiversity and indigenous livelihoods at risk [47]. Regenerative agriculture approaches are now helping to reverse deforestation trends and ensure the long-term health of Ethiopia’s ecosystems. The development of sustainable wild coffee and honey value chains through public-private partnerships in Kafa and Sheka provides a practical example of how cooperative-based approaches can support both conservation and livelihoods [46].

However, recent evidence also reveals the severe challenges facing coffee value chains in Ethiopia. The internal market in Ethiopia is largely determined by daily auctions run by the Ethiopian Commodity Exchange, but prices correlate closely with trends in global futures markets [48]. This means that fluctuations in financial markets in New York quickly feed through to prices at home. In 2025, high red cherry prices, rising production and logistics costs, and declining international prices significantly reduced margins across the entire value chain [48]. Ethiopia’s coffee exports fell 25 percent short of their volume target, with rising costs along the value chain eroding Ethiopia’s competitiveness in global markets [48]. This provides direct empirical support for our finding that coffee farmers face severe price volatility and lack the ability to plan investments.

The institutional mechanisms of value chain upgrading in Ethiopia’s coffee sector have been subject to recent analysis. A study found that product upgrading through the supply of higher quality coffee is mainly of interest for smallholders who benefit from successful cooperatives or outgrower schemes [49]. Cooperatives and unions have historically been able to exploit their economies of scale in separate export-oriented value chains, including for voluntary sustainability standards certification [49]. However, smallholder farmers also suffer from a lack of capital and imperfect capital markets, insufficient transport infrastructure, and information asymmetries. These factors undermine smallholder attempts to autonomously exploit new opportunities [48]. The government’s prioritization of coffee exports over meeting high domestic demand has led to extensive regulation and bureaucracy, creating rent-seeking opportunities that undermine transparency and fair pricing. This vicious circle is an obstacle to upgrading Ethiopian coffee value chains [49]. These findings are consistent with our observation that only 63.2% of coffee-honey households are cooperative members, leaving a substantial proportion outside the virtuous cycle. The implication is that cooperative interventions should include explicit outreach and inclusion strategies for poor and remote households.

### 4.3 Maize-livestock farming system

The maize-livestock system, comprising 14.9% of sampled households, is built on a foundational reinforcing loop where livestock provide traction and manure while crops provide feed. This finding aligns with and is substantially reinforced by recent research on integrated crop-livestock systems in African savannas. A study using coupled LUCIA and LIVSIM models found that crop-livestock integrated systems potentially allow sustainable intensification of crop and livestock production under climate variability in African savannas [50]. The integrated scenario allowed feeding maize straw and provided high-quality feed reserves from seasonally managed pastures. Livestock production was more resilient to land use change and drought with crop-livestock integration and intensively managed grazing [50]. However, without external fertilizer input, reasonable maize yields could only be achieved on fertile sites in the first years after conversion. This finding directly supports our observation that the biological synergy in maize-livestock systems is diminishing due to land fragmentation and soil fertility decline.

A study on nutrient recovery and recycling in Africa confirmed that manure nutrient recovery in Sub-Saharan Africa countries is strongly shaped by the realities of smallholder mixed crop-livestock systems [51]. Field experiments in Northern Ghana showed that compost application significantly improved maize productivity and soil health, with yield increases of 105.7% and 127.4% compared with control treatments [51]. The same paper contended as the groundnut-maize rotations with livestock achieved the highest carry-over of nutrients as manure, covering approximately one-third of the expected nitrogen, phosphorus, and potassium uptake by maize and reducing the demand for synthetic fertilizers. These findings highlight the potential of integrated soil fertility management approaches that combine organic and inorganic amendments to improve soil health and increase maize yields.

The soil mining trap (B1) we identified is the most critical balancing loop constraining the maize-livestock system. This finding aligns with Tittonell and Giller [3], who described soil nutrient mining as a primary driver of yield gaps in African smallholder systems. Our finding that households operating above approximately 2.3 TLU/ha experienced rapid declines in manure quality and soil fertility contradicts the optimistic assumption in some livestock development programs that more animals always mean more manure and therefore better soil fertility. Recent research confirms that the large-scale conversion of savanna grassland to maize fields is not recommended as it caused carbon losses from soil and vegetation [50]. This suggests the existence of a carrying capacity beyond which additional animals become counterproductive, a concept well established in rangeland ecology but rarely applied to smallholder mixed farming systems.

The feed-fuel competition loop (B2) is a novel contribution not emphasized in previous Ethiopian studies. Recent research confirms that the use of crop residue for fuel wood and animal feed creates significant trade-offs in Ethiopian smallholder systems. A study on biomass energy trade-offs in Ethiopia examined the allocation of biomass energy sources to domestic and productive uses [52]. Research in Central Ethiopia found that the main problems in preparing compost include using manure for fuel and crop residues for animal feeding, with 47.06% of farmers reporting this as a constraint [52]. As the Ethiopian energy demand urges for fuel options, it is essential to identify biomass fuels and estimate their energy potential [51]. The implication is that interventions that address energy poverty through biogas and improved cookstoves are not merely household welfare improvements but agricultural productivity interventions, challenging the sectoral silos that separate energy policy from agricultural policy.

### 4.4 Wheat-livestock farming system

The wheat-livestock system, comprising 19.2% of sampled households, features a nutrient recycling virtuous cycle where larger herds produce more manure, increasing soil organic matter and wheat yields. This finding aligns with Rufino et al. [4] on competing use of organic resources and with Vanlauwe et al. [5] on sustainable intensification. However, this study reveals a striking and unexpected finding: despite having the highest mean livestock holding (5.07 TLU) and the highest frequent extension contact (36.1%), the wheat-livestock system showed no significant productivity advantage over subsistence in the MESR results. This contradicts the expectation that higher asset levels and extension contact should translate into higher productivity. The divergence suggests that extension contact alone is insufficient when other constraints remain unaddressed. This finding challenges the common assumption in extension evaluation literature that increasing contact frequency automatically improves outcomes. It suggests that extension quality, content, and complementary services (credit, input access, disease management) may be more important than contact frequency.

The labor trade-off balancing loop (B1) is particularly binding in the wheat-livestock system. This finding aligns with Giller et al. [53] and Udo et al. [54], who identified labor trade-offs as a fundamental constraint in integrated crop-livestock systems. However, this finding contradicts the assumption in some mechanization literature that tractor adoption alone resolves labor constraints. Daum and Birner [55] argued that agricultural mechanization can transform smallholder agriculture by reducing labor requirements. In this study area, however, labor shortages are not only about tillage but also about weeding, harvesting, and daily livestock care. Custom-hire tractors address only one task, leaving other labor bottlenecks intact. The divergence likely arises from differences in the structure of labor constraints: in regions where tillage is the binding constraint, mechanization may indeed transform agriculture; in Jimma Zone, where multiple peak labor periods exist, tillage mechanization alone is insufficient. This suggests that mechanization programs should assess the specific labor bottlenecks in each context before promoting specific technologies.

The carrying capacity constraint (B2) aligns with Descheemaeker et al. [56]. The finding that wheat-livestock households had the highest mean livestock holding (5.07 TLU) but also reported animal health problems suggest that many households are operating beyond carrying capacity. This extends prior work by showing that the carrying capacity constraint is not only biophysical but also economic: households cannot reduce herd sizes because livestock serve as both productive assets and liquid savings. This dual role creates a path dependency where even when it would be economically rational to reduce herd sizes, households cannot do so without losing their primary form of savings.

The resource diversion loop (B3) extends Valbuena et al. [57] by showing that residue removal is not just a feed constraint but also a soil fertility constraint. The implication is that residue retention incentives (e.g., payments for ecosystem services, or linking residue retention to credit access) may be needed to break this loop. This finding contradicts the assumption in some agricultural economics literature that farmers will naturally adopt residue retention once they understand its benefits, suggesting instead that immediate cash needs systematically override long-term soil fertility considerations.

The most important finding from the wheat-livestock system is that credit access is only 8.3% in this system, the lowest among all four systems. This finding extends prior work by showing that credit constraints are not uniform across farming systems; wheat-livestock farmers face uniquely severe barriers.

### 4.5 Pathways for sustainability

Available literature clearly show as breaking the cycles of poverty and environmental degradation requires an integrated approach that simultaneously boosts productivity, improves human health, and restores ecological balance [58]. The same author contended as the foundational strategy is the promotion of nutrition-sensitive agroecology, which involves diversifying farms with climate-resilient, nutrient-dense crops like orange-fleshed sweet potato and drought-tolerant legumes. This directly addresses micronutrient deficiencies while spreading climatic and economic risk [59]. To counteract the soil mining prevalent in subsistence systems, integrated soil fertility management using compost, green manures, and trees is critical for rebuilding soil organic matter and enhancing water retention [58]. These efforts can be underpinned by social protection programs**,** which act as investments in human capital by ensuring basic food security. This prevents the distress sale of assets and provides the caloric energy needed for farmers to invest labor in their land, thereby strengthening the previously debilitating food-labour nexus [60].

For integrated crop-livestock systems, the focus must be on enhancing the quality and reliability of the biomass cycle. In the maize-livestock system**,** the soil fertility-feed gap nexus can be decoupled by integrating dual-purpose legumes. These legumes fix atmospheric nitrogen while providing high-protein fodder, which enhances livestock nutrition and, consequently, manure output [60,61]. This can be paired with feed resource optimization, such as treating stover with urea to improve digestibility or creating small-scale silage banks to stabilize dry-season feed and manure production [62]. Furthermore, promoting sustainable energy alternatives like biogas digesters reduces the reliance on crop residues for fuel, allowing more biomass to be cycled back into the soil.

The sustainable intensification of the wheat-livestock system requires strategies that enhance input efficiency, alleviate labor pressures, and manage biotic threats. This involves precision agronomy and financial instruments**,** such as site-specific nutrient management and input credit schemes, to de-risk the high-cost nature of production [63]. Strategic mechanization via custom-hire services for planting and harvesting is crucial to break the peak-season labor bottleneck, freeing up household labor for other tasks [55]. To protect yields, an integrated pest and disease management approach mandating disease-resistant varieties and promoting early warning systems is essential for proactive threat management [64].

Finally, securing the future of the coffee-honey system hinges on building climate resilience and stabilizing incomes through deeper ecological and economic integration. The foremost strategy is a transition to diversified, climate-smart agroforestry. A multi-layered canopy of native shade trees buffers against temperature extremes, conserves moisture, and provides a stable habitat for the pollinating insects upon which coffee yields depend [65]. Actively promoting beekeeping bolsters this pollination service while diversifying income. To counter market volatility, value chain integration and diversification are critical. Supporting certifications for shade-grown or organic coffee can secure price premiums, while developing markets for honey, fruits, and timber from the agroforestry system provides a crucial buffer against coffee price crashes, creating a more resilient and profitable enterprise [66].

## 5 Conclusions and recommendations

### 5.1 Summary of empirical findings and scientific contributions

This study examined the dynamics, challenges, and sustainability pathways for four coexisting farming systems in Jimma Zone, Southwestern Ethiopia, using a system dynamics modeling approach based on data from 375 households, 8 focus group discussions, and 21 key informant interviews. The empirical findings reveal three core patterns that together constitute the scientific contribution of this research. First, four distinct farming system typologies were identified to coexist in the study area. These are; subsistence (32.5% of households), coffee-honey (33.3%), maize-livestock (14.9%), and wheat-livestock (19.2%). These systems are not randomly distributed but are closely associated with agro-ecological zones: wheat-livestock in highlands (Dedo), coffee-honey in midlands (Gera), and maize-livestock in lowlands (Sekoru), with subsistence systems appearing across all zones. This typology confirms the agro-ecological determinism of farming system distribution while revealing that subsistence farming persists across all zones as a residual category for the most resource-constrained households. Second, each system is characterized by distinct self-reinforcing constraints that operate through specific causal feedback loops.

The subsistence system is trapped in a food-labor nexus where food insecurity (reported by 94.3% of subsistence households) weakens labor capacity, further reducing productivity and creating a reinforcing poverty trap that cannot be broken by input subsidies alone. The coffee-honey system faces a deforestation-pollination-price triad: forest cover has declined from an estimated 40–60% to 15–25%, climate change is perceived by 80.8% of coffee farmers, and global price volatility forces disinvestment in bee-friendly practices, creating a downward spiral that undermines both coffee and honey productivity. The maize-livestock system is constrained by a soil mining trap where overgrazing (2.3 TLU/ha versus recommended 0.8 TLU/ha) depletes soil fertility, and by feed-fuel competition where 43% of households using stover for cooking reduces livestock condition by 27%, breaking the biological synergy that historically sustained productivity. The wheat-livestock system faces a labor trade-off where 61.3% of households report peak-season labor shortages, and credit access is only 8.3% (the lowest among all systems), preventing investment in improved inputs despite the highest extension contact (36.1%). Third, a common poverty trap operates across all systems: low productivity limits marketable surplus and cash income, which prevents investment in productivity-enhancing assets, keeping households in a low-equilibrium state vulnerable to shocks. However, the specific mechanisms of this trap vary systematically by system, implying that one-size-fits-all interventions are unlikely to succeed.

The scientific value added of this paper is threefold. First, this study provides the first empirical application of formal causal loop diagramming to map feedback dynamics across coexisting farming systems in an Ethiopian context. While previous research has either examined individual farming systems in isolation, treated system choice as a static outcome, or provided descriptive case studies without formal causal modeling, this study makes explicit the causal relationships and feedback loops that govern each system’s behavior over time. The systematic four-phase protocol variable identification, causal relationship extraction, loop construction, and participatory validation provides a transparent and replicable methodology that can be applied in other contexts. Second, this study contributes to the ongoing debate in agricultural development between technology-driven and systems-oriented approaches by demonstrating that the appropriate intervention strategy depends on the specific causal architecture of each system. The finding that the same poverty trap operates through different mechanisms across systems challenges the assumption that generic interventions can be applied uniformly across diverse smallholder contexts.

The identification of system-specific leverage points the food-labor nexus in subsistence, the deforestation-pollination link in coffee-honey, the feed-fuel competition in maize-livestock, and the labor-credit-pest complex in wheat-livestock provides empirical evidence that effective policy design requires understanding the unique causal feedback structures of each system. Third, this study contributes to social-ecological systems theory by operationalizing the SES framework through measurable variables and translating theoretical components into empirically grounded causal loop diagrams. This demonstrates how the SES framework can be applied beyond conceptual analysis to generate practical policy insights, bridging the gap between systems theory and agricultural development practice.

### 5.2 Applicability of findings and policy implications

The findings of this study have direct applicability for policy design and agricultural development programming in Ethiopia and other smallholder farming contexts. Based on the causal loop analysis and feasibility assessment, the following interventions are suggested as potential pathways for breaking the identified constraint cycles. These are proposed implications derived from the system dynamics model, not empirically demonstrated outcomes, as no interventions were tested in this study.

For the subsistence system, bundled nutrition-sensitive social protection is suggested to address the food-labor nexus. This could involve integrating the Productive Safety Net Program with agricultural input support and nutrition interventions to simultaneously address food insecurity and labor capacity constraints. The causal loop diagram reveals that providing inputs alone to malnourished households will have limited impact because labor capacity is the binding constraint; therefore, nutrition support must precede or accompany input provision.

For the maize-livestock system, promoting dual-purpose legumes and feed optimization is suggested to close the soil fertility-feed gap. This includes introducing leguminous crops that fix nitrogen while providing high-protein fodder, treating stover with urea to improve digestibility, and promoting biogas or improved cookstoves to reduce stover burning. The causal loop diagram shows that the soil mining trap operates through the interaction of overgrazing, declining manure quality, and feed scarcity, so interventions must address all three elements simultaneously.

For the wheat-livestock system, facilitating custom-hire mechanization services and developing wheat-specific credit products with harvest-aligned repayment schedules is suggested to address labor and credit constraints. The causal loop diagram reveals that labor shortages during peak seasons and the complete lack of formal credit for wheat farmers are the binding constraints, so interventions must focus on reducing labor bottlenecks and providing financial services tailored to wheat production cycles.

For the coffee-honey system, promoting climate-smart agroforestry with optimal shade levels (30–50% cover, not full sun) and strengthening cooperative-based price bargaining is suggested to buffer climate and market shocks. The causal loop diagram shows that deforestation and price volatility create reinforcing downward spirals, so interventions must simultaneously address ecological management and market integration.

These interventions are sequenced by feasibility: short-term (0–2 years) includes extension reorientation and mobile market information; medium-term (2–5 years) includes biogas subsidies and credit product reform; long-term (>5 years) includes road infrastructure and landscape-scale forest restoration. The causal loop diagrams provide a decision-support tool that enables policymakers to trace how each intervention would propagate through the system, anticipate potential unintended consequences, and identify the sequence of interventions most likely to generate virtuous cycles. For example, the analysis reveals that providing credit to wheat-livestock farmers without addressing labor shortages will have limited impact because labor, not capital, is the binding constraint during peak seasons. Similarly, providing improved cookstoves to maize-livestock households without addressing overgrazing will have limited impact on soil fertility because manure quality and quantity are constrained by overgrazing, not just by residue availability. This systems-level understanding enables more cost-effective and targeted resource allocation, avoiding the common pattern of implementing isolated interventions that fail to address the underlying causal structures.

### 5.3 Limitations

Several limitations should be considered when interpreting these findings. First, the cross-sectional design cannot capture dynamic transitions between farming systems over time; longitudinal panel data would be needed to test the causal loop structures empirically. Second, the sample is heavily male-dominated (91.5% male respondents); findings primarily reflect male household head perspectives, and gender-specific analysis is recommended for future research. Although the response rate was high (>95%), non-response bias cannot be entirely ruled out, particularly among female-headed households. Third, the study is context-specific to Jimma Zone; generalizability to other Ethiopian regions or sub-Saharan African countries requires further validation. Fourth, as noted above, intervention outcomes are proposed based on causal loop analysis, not empirically demonstrated; pilot studies are needed to test the suggested pathways.

### 5.4 Future research directions

Four future research directions are recommended. First, longitudinal panel data should track how households transition between farming systems over time and test the causal mechanisms identified in the CLDs. Second, intervention pilots should test the sequenced recommendations (e.g., nutrition-first vs input-first in subsistence systems) to estimate causal effects on productivity and household welfare. Third, gender-disaggregated studies with quotas (minimum 30% female respondents) should examine women’s roles in decision-making, labor allocation, and income control across systems. Fourth, comparative studies across other Ethiopian zones should assess the generalizability of the causal loop structures identified here and validate the quantified thresholds (e.g., overgrazing carrying capacity, optimal shade cover) in different agro-ecological contexts.

## Supporting information

S1 File Model audit trail for causal loop diagram construction. (DOCX)

S2 File Triangulation matrix for CLD variables and causal links. (DOCX)

S3 File Semi-structured interview guide. (DOCX)

S4 File Compliance with CROSS and COREQ guidelines. (DOCX)
