# Supplementary material for: Farming system dynamics and sustainability pathways in three districts of Jimma zone, Southwestern Ethiopia
Source: PLoS One. 2026 Oct 28;21(10):e0359541. doi: 10.1371/journal.pone.0359541 (PMC13716233; doi:10.1371/journal.pone.0359541)
Supplement: Supplementary file 1 [file pone.0359541.s001.docx]

**Supplementary File S1**

**Model Audit Trail for Causal Loop Diagram Construction**

**Study Title: Systems Dynamics Analysis of Interlinked Farming System and Sustainability Pathways in the Jimma Zone, Southwestern Ethiopia**

**Date:** May 10/2026

**Version:** Final

**Table of Contents**

Contents

[Supplementary File	1](#_Toc228979905)

[Model Audit Trail for Causal Loop Diagram Construction	1](#_Toc228979906)

[Table of Contents	2](#_Toc228979907)

[1. Introduction and Purpose	2](#_Toc228979908)

[2. Overview of Data Sources	3](#_Toc228979909)

[3. Phase 1 Audit: Variable Identification	3](#_Toc228979910)

[3.1 Initial Code Lists (Independent Coders)	4](#_Toc228979911)

[3.2 Consensus Resolution Log	4](#_Toc228979912)

[3.3 Variables Excluded with Reasons	5](#_Toc228979913)

[3.4 Final Retained Variables by Farming System	5](#_Toc228979914)

[4. Phase 2 Audit: Causal Relationship Extraction	7](#_Toc228979915)

[4.1 Causal Statement Extraction Table (Sample)	7](#_Toc228979916)

[4.2 Links Excluded with Reasons	8](#_Toc228979917)

[4.3 Final Retained Links by Farming System	9](#_Toc228979918)

[5. Phase 3 Audit: CLD Construction	9](#_Toc228979919)

[5.1 Loop Polarity Calculations	9](#_Toc228979920)

[5.2 Cross-System Comparison Matrix	10](#_Toc228979921)

[6. Phase 4 Audit: Validation Outcomes	10](#_Toc228979922)

[6.1 Member Checking (Participant Validation) Log	10](#_Toc228979923)

[6.2 Expert Validation Log	11](#_Toc228979924)

[6.3 Post-Validation Modifications	12](#_Toc228979925)

[7. Declaration of Completeness	13](#_Toc228979926)

# 1. Introduction and Purpose

This audit trail documents all decisions made during the construction of causal loop diagrams (CLDs) for the four farming systems (subsistence, coffee-honey, maize-livestock, and wheat-livestock) in Jimma Zone, Ethiopia. Following best practices for reproducible system dynamics modeling (Sterman, 2000; Videira et al., 2017), this trail records:

- All variables and causal links that were proposed
- All variables and links that were excluded and the reasons for exclusion
- All modifications made during participant and expert validation
- The final retained model elements

This document ensures that the modeling process is transparent, auditable, and replicable by other researchers.

# 2. Overview of Data Sources

| **Data Source** | **Number** | **Participant/Household Count** | **Purpose in CLD Construction** |
| --- | --- | --- | --- |
| Focus Group Discussions (FGDs) | 8 | 72 participants (8-10 per FGD) | Variable identification; causal statement extraction; loop validation |
| Key Informant Interviews (KIIs) | 21 | 21 informants | Variable identification; causal statement extraction; cross-checking |
| Household Survey | 375 | 375 households | Quantitative validation of variable importance (descriptive statistics) |
| Field Observations | 9 kebeles | N/A | Contextual grounding; verification of observable links |

# 3. Phase 1 Audit: Variable Identification

## 3.1 Initial Code Lists (Independent Coders)

Two researchers (Coder A and Coder B) independently coded 30% of transcripts (randomly selected: 2 FGDs and 6 KIIs).

| Coder | Variables Identified (Initial) | Unique to Coder | Overlap with Other Coder |
| --- | --- | --- | --- |
| Coder A | 52 | 11 | 41 |
| Coder B | 48 | 7 | 41 |
| **Consensus after discussion** | **47** | N/A | **41 (87% agreement)** |

*Intercoder agreement rate: 87% (exceeds the >80% threshold specified in methodology).*

## 3.2 Consensus Resolution Log

Disagreements between coders were resolved through discussion with a third researcher. Below are examples of resolved disagreements:

| **Variable** | **Coder A Classification** | **Coder B Classification** | **Resolution** | **Justification** |
| --- | --- | --- | --- | --- |
| "Coffee cherry quality" | Separate variable | Merged with "coffee yield" | Merged as "coffee yield (quality-adjusted)" | Participants used the terms interchangeably; no distinct causal links unique to quality alone |
| "Time spent walking to market" | Separate variable | Part of "market access" | Retained as separate variable | Participants distinguished between distance (physical) and price/market information (institutional); different causal links |
| "Family harmony" | Included | Excluded (not system-relevant) | Excluded | Mentioned by only 2 participants; no causal links to farming outcomes consistently reported |

## 3.3 Variables Excluded with Reasons

A total of **13 variables** were proposed during open coding but excluded after applying inclusion criteria (C1: ≥5 participants, C2: causal link present, C3: policy relevance).

| Excluded Variable | Proposed By (Source) | Reason for Exclusion | Criterion Failed |
| --- | --- | --- | --- |
| "Specific fertilizer brand (e.g., DAP vs. Urea)" | KII #4 (agricultural expert) | Mentioned by only 2 participants; no system-level relevance | C1, C2 |
| "Name of local market center" | FGD #5 (coffee-honey) | No causal links to system outcomes | C2 |
| "Neighbor's farming practice" | FGD #2 (subsistence) | Mentioned by only 1 participant; too vague | C1 |
| "Color of soil" | FGD #7 (maize-livestock) | Participant observation but no reported causal effect on yields | C2 |
| "Distance to school" | KII #12 (kebele administrator) | Not related to farming system dynamics | C2, C3 |
| "Number of wives" | FGD #3 (wheat-livestock) | Cultural variable with no causal link to productivity reported; mentioned by 2 participants | C1, C2 |
| "Type of cooking stove" | FGD #6 (coffee-honey) | Mentioned by 3 participants but no clear causal link to farming system | C2 |
| "Price of teff (non-study crop)" | KII #18 (cooperative leader) | Teff is not a primary crop in the study area; mentioned by 1 participant | C1 |
| "Health center distance" | FGD #1 (subsistence) | Social service variable not directly linked to farming decisions | C2 |
| "Mobile phone ownership" | FGD #4 (maize-livestock) | Mentioned by 4 participants but no consistent causal link to productivity; some used for price info, others not | C2 (insufficient causal evidence) |
| "Celebration expenses (holidays)" | KII #7 (farmer) | Mentioned by 2 participants as affecting savings, but link to farming not established | C1, C2 |
| "Soil color (red vs. black)" | FGD #8 (wheat-livestock) | Observable but no reported productivity difference; participants said "both soils are same for wheat" | C2 |
| "Number of beehives (old style vs. modern)" | FGD #5 (coffee-honey) | Mentioned by 3 participants; merged into "bee health/hive productivity" as the causal variable | C2 (merged) |

## 3.4 Final Retained Variables by Farming System

After applying inclusion criteria and resolving disagreements, **47 variables** were retained across the four farming systems. The distribution is as follows:

| Farming System | Number of Retained Variables | Unique to This System | Shared with Other Systems |
| --- | --- | --- | --- |
| Subsistence | 22 | 8 | 14 |
| Coffee-honey | 28 | 12 | 16 |
| Maize-livestock | 24 | 9 | 15 |
| Wheat-livestock | 25 | 10 | 15 |

**Variables appearing in all four systems (core variables):**

- Soil fertility
- Labor availability
- Household income
- Crop yield
- Climate change impact
- Market access
- Extension contact
- Credit access

**Variables appearing in three of four systems:**

- Livestock health (absent from coffee-honey)
- Land size (absent from wheat-livestock)
- Input access (absent from subsistence)

# 4. Phase 2 Audit: Causal Relationship Extraction

## 4.1 Causal Statement Extraction Table (Sample)

Below is a sample of the extraction table used to record causal statements.

| Statement ID | Source | Original Quote (translated) | Independent Variable | Dependent Variable | Direction | Confidence | Time Lag |
| --- | --- | --- | --- | --- | --- | --- | --- |
| S-001 | FGD #1 (Subsistence), P3 | "When the rains are late, our maize does not grow well" | Rainfall timing | Maize yield | (+) | High | Same season |
| S-002 | FGD #1 (Subsistence), P5 | "If we don't have enough food, the children are too weak to help in the fields" | Food security | Labor availability | (+) | High | Within months |
| S-003 | FGD #1 (Subsistence), P7 | “When we have no money for seed, we plant the old seed and get less harvest" | Financial capacity | Input quality | (+) | High | Same season |
| S-004 | FGD #2 (Coffee-honey), P2 | "More shade trees means more moisture in the soil, and the coffee gives better" | Tree cover | yield | (+) | High | Same season |
| S-005 | FGD #2 (Coffee-honey), P6 | "When we cut trees for more coffee land, the bees disappear" | Deforestation | Bee health | (-) | High | 1-2 years |
| S-006 | FGD #3 (Maize-livestock), P4 | "The more animals we have, the more manure we get, and the maize grows taller" | Livestock herd size | Manure availability | (+) | High | Same season |
| S-007 | FGD #3 (Maize-livestock), P8 | "When we use maize stover for cooking, the cows have nothing to eat in dry season" | Stover for fuel | Feed availability | (-) | Medium | Within months |
| S-008 | FGD #4 (Wheat-livestock), P3 | "If we don't have fertilizer, the wheat gives very little" | Input access | Wheat yield | (+) | High | Same season |
| S-009 | KII #5 (Ag expert) | "Disease outbreaks in wheat are worse when farmers plant the same variety year after year" | Crop diversity | Pest/disease pressure | (-) | High | 2-3 years |
| S-010 | KII #11 (Coop leader) | "Cooperatives help farmers get better prices because they can sell together" | Cooperative strength | Bargaining power | (+) | High | Immediate |

## 4.2 Links Excluded with Reasons

A total of 42 proposed links were excluded after failing retention criteria (A: ≥3 participants, B: ≥2 data sources).

| Link ID | Proposed Link | Direction | Source(s) | Reason for Exclusion |
| --- | --- | --- | --- | --- |
| L-001 | Coffee yield → Child school attendance | (+) | FGD #2 only (1 source) | Failed B (<2 data sources) |
| L-002 | Road quality → Coffee price | (+) | KII #4 only (1 source) | Failed B (<2 data sources) |
| L-003 | Manure quality → Human health | (+) | FGD #3, P2 only (1 participant) | Failed A (<3 participants) |
| L-004 | Age of household head → Coffee yield | (-) | FGD #2 (2 participants) | Failed A (only 2 participants) |
| L-005 | Rainfall → Migration | (-) | FGD #1 (2 participants) | Failed A (only 2 participants) |
| L-006 | Education → Off-farm income | (+) | KII #12 only (1 source) | Failed B (<2 data sources) |
| L-007 | Livestock sales → Children's marriage expenses | (+) | FGD #3 (1 participant) | Failed A (<3 participants) |
| L-008 | Coffee price → Local construction activity | (+) | KII #18 (1 participant) | No plausible causal link to farming system outcomes |
| L-009 | Fertilizer use → Weed population | (+) | FGD #8 (2 participants) | Failed A (<3 participants); contradictory reports |
| L-010 | Distance to water → Time allocation | (-) | FGD #6 (2 participants) | Failed A (<3 participants) |

## 4.3 Final Retained Links by Farming System

After applying retention criteria, the following number of causal links was retained for CLD construction:

| **Farming System** | **Proposed Links (Phase 2)** | **Retained Links** | **Retention Rate (%)** |
| --- | --- | --- | --- |
| Subsistence | 48 | 38 | 79% |
| Coffee-honey | 62 | 51 | 82% |
| Maize-livestock | 58 | 47 | 81% |
| Wheat-livestock | 55 | 44 | 80% |
| **Total** | **223** | **180** | **81%** |

# 5. Phase 3 Audit: CLD Construction

## 5.1 Loop Polarity Calculations

For each identified loop, polarity was calculated as the product of all link polarities. Below is a sample calculation:

**Loop L-SUB-01 (Subsistence System): "Food-Labor Nexus"**

| **Link** | **Polarity** |
| --- | --- |
| Food security → Labor availability | (+) |
| Labor availability → Crop yield | (+) |
| Crop yield → Food security | (+) |
| Product | (+) × (+) × (+) = (+) |
| Loop Type | Reinforcing (R) (vicious cycle: low food → low labor → low yield → low food) |

**Loop L-ML-03 (Maize-Livestock System): "Soil Mining Trap"**

| **Link** | **Polarity** |
| --- | --- |
| Crop yield → Stover availability | (+) |
| Stover availability → Feed availability | (+) |
| Feed availability → Manure production | (+) |
| Manure production → Soil fertility | (+) |
| Soil fertility → Crop yield | (+) |
| Product | (+) × (+) × (+) × (+) × (+) = (+) |
| Loop Type | Reinforcing (R) (vicious cycle: low yield → low stover → low feed → low manure → low fertility → low yield) |

**5.2 Cross-System Comparison Matrix**

Variables were compared across the four systems to identify common and system-specific elements.

| Variable | Subsistence | Coffee-Honey | Maize-Livestock | Wheat-Livestock | Count |
| --- | --- | --- | --- | --- | --- |
| Soil fertility | ✓ | ✓ | ✓ | ✓ | 4 |
| Labor availability | ✓ | ✓ | ✓ | ✓ | 4 |
| Household income | ✓ | ✓ | ✓ | ✓ | 4 |
| Crop yield | ✓ | ✓ | ✓ | ✓ | 4 |
| Climate change impact | ✓ | ✓ | ✓ | ✓ | 4 |
| Market access | ✓ | ✓ | ✓ | ✓ | 4 |
| Extension contact | ✓ | ✓ | ✓ | ✓ | 4 |
| Credit access | ✓ | ✓ | ✓ | ✓ | 4 |
| Livestock health | ✗ | ✗ | ✓ | ✓ | 2 |
| Tree cover/forest | ✗ | ✓ | ✗ | ✗ | 1 |
| Bee health/pollinators | ✗ | ✓ | ✗ | ✗ | 1 |
| Manure quality | ✗ | ✗ | ✓ | ✓ | 2 |
| Stover availability | ✗ | ✗ | ✓ | ✓ | 2 |

# 6. Phase 4 Audit: Validation Outcomes

**6.1 Member Checking (Participant Validation) Log**

A one-day validation workshop was held with 24 participants (6 from each farming system). Below is the validation log:

| **Validation Activity** | **Criterion** | **Result** | **Action Taken** |
| --- | --- | --- | --- |
| Variable verification (Subsistence) | ≥80% agreement | 87% agreed | No changes |
| Variable verification (Coffee-honey) | ≥80% agreement | 91% agreed | No changes |
| Variable verification (Maize-livestock) | ≥80% agreement | 85% agreed | No changes |
| Variable verification (Wheat-livestock) | ≥80% agreement | 88% agreed | No changes |
| Link verification: "Rainfall → Crop yield" | ≥70% agreement | 96% agreed | Retained |
| Link verification: "Credit access → Input purchase" | ≥70% agreement | 79% agreed | Retained |
| Link verification: "Tree cover → Coffee yield" | ≥70% agreement | 92% agreed | Retained |
| Link verification: "Stover for fuel → Livestock health" | ≥70% agreement | 68% agreed (below threshold) | Removed (see Section 6.3) |
| Link verification: "Cooperative membership → Market price" | ≥70% agreement | 83% agreed | Retained |
| Link verification: "Wealth status → Risk tolerance" | ≥70% agreement | 54% agreed (below threshold) | Removed (see Section 6.3) |
| Loop verification (all loops) | Concrete example provided | 24/24 loops had examples | All retained |
| Loop naming (Afan Oromo) | At least one name per loop | 8 names collected | Incorporated into final CLDs |

**Participant-generated loop names (Afan Oromo with English translation):**

| Loop ID | Participant Name (Afan Oromo) | English Translation |
| --- | --- | --- |
| L-SUB-01 | "Jireenya harkaafi afaanii" | Hand to Mouth life (food-labor nexus) |
| L-CH-02 | "Muka lafaaf, Kanniisota mukaaf" | Trees for land, bees for land (deforestation-pollination loop) |
| L-ML-03 | "Biyyee nyaachuu fi bineensota nyaachisuu" | Eating the soil and feeding the animals (soil mining trap) |
| L-WL-01 | "Yeroo qotiyye fi midhaan wal lolu" | When crop and livestock fight (labor trade-off) |

## 6.2 Expert Validation Log

A panel of 5 experts reviewed the draft CLDs:

| Expert ID | Expertise | Agreement with Variable Set | Agreement with Link Set | Agreement with Loop Structure | Comments |
| --- | --- | --- | --- | --- | --- |
| E1 | Agricultural economist | Yes | Yes (with 1 suggested addition) | Yes | Suggested adding "off-farm income diversification" to subsistence CLD |
| E2 | Agricultural economist | Yes | Yes | Yes | No major comments |
| E3 | System dynamics modeler | Yes | Yes (with 2 suggestions) | Yes (with 1 suggestion) | Suggested simplifying loop labels; added note on polarity consistency |
| E4 | Senior agricultural researcher (Ethiopia) | Yes | Yes | Yes | Confirmed local relevance |
| E5 | Senior agricultural researcher (Ethiopia) | Yes (with 1 suggestion) | Yes | Yes | Suggested adding "soil erosion" explicitly to maize-livestock CLD |

**Consensus: 5/5 experts agreed on final CLDs after incorporating modifications (see Section 6.3).**

## 6.3 Post-Validation Modifications

Based on participant and expert feedback, the following modifications were made to the final CLDs:

| Modification ID | Original Element | Change Made | Source of Feedback | Justification |
| --- | --- | --- | --- | --- |
| M-001 | Link: Stover for fuel → Livestock health | **Removed** | Member checking (68% agreement, below 70% threshold) | Participants reported that the link exists but is weak; not strong enough to retain as a direct causal link |
| M-002 | Link: Wealth status → Risk tolerance | **Removed** | Member checking (54% agreement) | Participants reported wealth affects many things but not consistently risk tolerance in farming decisions |
| M-003 | Variable: "Off-farm income diversification" | **Added** to subsistence CLD | Expert E1 | Recognized as important pathway for breaking poverty cycle in subsistence system |
| M-004 | Link: Off-farm income → Financial capacity | **Added** to subsistence CLD | Expert E1 | Completes the new variable's causal connections |
| M-005 | Variable: "Soil erosion" | **Added** to maize-livestock CLD | Expert E5 | Important missing link in the soil fertility-feed gap loop |
| M-006 | Link: Overgrazing → Soil erosion | **Added** to maize-livestock CLD | Expert E5 | Completes the erosion pathway in soil mining trap |
| M-007 | Loop label: "Soil mining trap" | **Simplified** from "Organic matter depletion - nutrient mining complex" | Expert E3 | Improved clarity for readers |
| M-008 | Loop polarity check (all loops) | **Verified consistent** | Expert E3 | All 25 loops confirmed with correct R/B labeling |

# 7. Declaration of Completeness

I, Tamiru Chalchisa, as the corresponding author, declare that this audit trail documents all substantive decisions made during the construction of causal loop diagrams for this study. To the best of my knowledge:

1. All variables proposed during open coding are documented in Section 3, with clear reasons for inclusion or exclusion.
2. All causal links extracted from transcripts are documented in the accompanying Excel file (Supplementary File S2), with excluded links and reasons provided in Section 4.2.
3. All validation feedback from participants and experts is documented in Section 6, with all resulting modifications recorded.
4. No variables, links, or loops were omitted from this audit trail for reasons of convenience or presentational preference.

**Date:** ________________

**Signature:** ________________

**Name:** Tamiru Chalchisa

**Corresponding Author Email:** tamiruchalchisa2005@gmail.com
