# Supplementary material for: Farming system dynamics and sustainability pathways in three districts of Jimma zone, Southwestern Ethiopia
Source: PLoS One. 2026 Oct 28;21(10):e0359541. doi: 10.1371/journal.pone.0359541 (PMC13716233; doi:10.1371/journal.pone.0359541)
Supplement: Supplementary file 2 [file pone.0359541.s002.docx]

**Supplementary File S2: Triangulation Matrix for CLD Variables and Causal Links**

**Table S2: Triangulation Matrix for CLD Variables and Causal Links**

| **CLD Element** | **Farming System** | **Qualitative Evidence** | **Quantitative Indicator** | **Convergence Status** | **Resolution**  **Action** |
| --- | --- | --- | --- | --- | --- |
| **VARIABLE: Soil fertility** | All four systems | Mentioned in 7/8 FGDs (87.5%); 54 participant quotes. Example quote – FGD #3 (Maize-livestock): "The land is tired now; it doesn't give like before" | 86.2% of households (n=323) reported perceived decline in soil fertility over past 10 years; mean soil organic carbon: 1.6% (SD=0.7) | **Full convergence** | Retained as core variable |
| **VARIABLE: Labor availability** | All four systems | Mentioned in all 8 FGDs (100%); 67 participant quotes. *Example quote – FGD #1 (Subsistence): "When people are hungry, they cannot work hard"* | Mean family size: 5.61 persons; 61.3% of households (n=230) report labor shortage during peak seasons (weeding/harvest) | **Full convergence** | Retained as core variable |
| **VARIABLE: Household income** | All four systems | Mentioned in 8/8 FGDs (100%); 89 participant quotes. Example quote – FGD #5 (Coffee-honey): "When coffee prices are good, we can buy food and seed" | Mean annual farm income: 32,890 ETB (SD=34,210); off-farm income: 3,890 ETB (SD=6,120) | **Full convergence** | Retained as core variable |
| **VARIABLE: Credit access** | All four systems | Mentioned in 6/8 FGDs (75%); 31 participant quotes. Example quote – FGD #4 (Wheat-livestock): "If we could get loans, we would buy better seed and fertilizer" | Only 16.8% of households (n=63) accessed formal credit in past 12 months; among those, 74% purchased improved inputs | **Full convergence** | Retained as core variable |
| **VARIABLE: Extension contact** | All four systems | Mentioned in 5/8 FGDs (62.5%); 28 participant quotes. *Example quote – KII #4 (Development agent): "Farmers who get regular visits adopt better practices"* | Only 14.9% of households (n=56) have frequent extension contact; 57.3% occasional; 27.7% rare or none | **Full convergence** | Retained as core variable |
| **VARIABLE: Tree cover / Forest** | Coffee-honey only | Mentioned in 3/3 coffee-honey FGDs (100%); 28 participant quotes. *Example quote – FGD #2: "Shade trees keep coffee healthy, but we have cut many for more land"* | Estimated canopy cover declined from historical 40-60% to current 15-25% in study area; 80.8% of coffee farmers report climate change impacts linked to deforestation | **Full convergence** | Retained as system-specific variable |
| **VARIABLE: Bee health / Pollinators** | Coffee-honey only | Mentioned in 3/3 coffee-honey FGDs (100%); 24 participant quotes. *Example quote – FGD #2: "When forest is gone, bees disappear and honey yield drops"* | Honey yield averaged 8.2 kg/hive/year (range 2-15); coffee yield positively correlated with proximity to forest patches (r=0.34, p<0.01) | **Full convergence** | Retained as system-specific variable |
| **VARIABLE: Livestock health** | Maize-livestock, Wheat-livestock | Mentioned in 4/4 mixed system FGDs (100%); 35 participant quotes. *Example quote – FGD #3: "Weak oxen cannot plow well, and manure is poor"* | Mean livestock body condition score: 2.4/5 (SD=0.8); households with higher TLU had lower condition scores (r=-0.29, p<0.01) | **Full convergence** | Retained for mixed systems |
| **LINK: Manure → Soil fertility (+)** | Maize-livestock, Wheat-livestock | Mentioned in 6/8 FGDs (75%); 42 participant quotes. *Example quote – FGD #3: "More manure means better maize, everyone knows this"* | Households applying manure had 28% higher maize yield (4.2 vs 3.3 qu/ha, t=4.21, p<0.001); correlation r=0.52 | **Full convergence** | Retained as positive (+) link |
| **LINK: Crop yield → Household income (+)** | All four systems | Mentioned in 8/8 FGDs (100%); 112 participant quotes. *Example quote – FGD #2: "Good harvest means we can sell and buy what we need"* | Correlation between crop yield and farm income: r=0.67 (p<0.001); top quartile yield households had 3.2x higher income | **Full convergence** | Retained as positive (+) link |
| **LINK: Income → Input purchase (+)** | All four systems | Mentioned in 7/8 FGDs (87.5%); 56 participant quotes. *Example quote – FGD #4: "When we have cash, we buy improved seed first"* | Households in top income tercile spent 4x more on inputs (2,100 vs 520 ETB/year, p<0.001) | **Full convergence** | Retained as positive (+) link |
| **LINK: Deforestation → Bee health (-)** | Coffee-honey only | Mentioned in 3/3 coffee-honey FGDs (100%); 21 participant quotes. *Example quote – FGD #2: "When we cut trees, bees leave – no honey"* | Households with >50% tree cover reported 2.3x higher honey yield (12.5 vs 5.4 kg/hive); Spearman's ρ = -0.47 | **Full convergence** | Retained as negative (-) link |
| **LINK: Overgrazing → Soil fertility (-)** | Maize-livestock, Wheat-livestock | Mentioned in 5/8 FGDs (62.5%); 34 participant quotes. *Example quote – FGD #7: "Too many cows on small land – the ground becomes hard and barren"* | Livestock density averaged 2.3 TLU/ha vs recommended 0.8 TLU/ha; higher density correlated with lower soil organic matter (r=-0.41, p<0.001) | **Full convergence** | Retained as negative (-) link |
| **LINK: Stover for fuel → Livestock health (-)** | Maize-livestock only | Mentioned in 4/8 FGDs (50%); 19 participant quotes. *Example quote – FGD #3: "We burn stalks for cooking, then cows have nothing to eat in dry season"* | 43% of households use crop residues as primary cooking fuel; among these, livestock body condition was 27% lower (2.1 vs 2.9/5, p<0.01) | **Full convergence** | Retained as negative (-) link |
| **LINK: Credit access → Input purchase (+)** | All four systems | Mentioned in 5/8 FGDs (62.5%); 31 participant quotes. *Example quote – FGD #4: "If we could get loans, we would buy better seed"* | Households with credit access were 3.4x more likely to purchase improved inputs (74% vs 22%, χ²=42.3, p<0.001) | **Full convergence** | Retained as positive (+) link |
| **LINK: Extension contact → Technology adoption (+)** | All four systems | Mentioned in 5/8 FGDs (62.5%); 28 participant quotes. *Example quote – KII #4: "Farmers we visit regularly use more improved practices"* | Households with frequent extension contact had 2.8x higher adoption rate of improved seed (68% vs 22%, χ²=38.1, p<0.001) | **Full convergence** | Retained as positive (+) link |
| **LINK: Global coffee price → Farm profit (+)** | Coffee-honey only | Mentioned in 3/3 coffee-honey FGDs (100%); 18 participant quotes. *Example quote – FGD #5: "When world price drops, we get nothing – can't even buy food"* | 71% of coffee-farming households reported reducing investment when prices drop; price volatility index correlated with annual income variation (r=0.38, p<0.01) | **Full convergence** | Retained as positive (+) link |
| **LINK: Food security → Labor availability (+)** | Subsistence only | Mentioned in 2/2 subsistence FGDs (100%); 14 participant quotes. *Example quote – FGD #1: "When children are hungry, they cannot help in the fields"* | Households reporting food insecurity (94.3%) had 32% lower labor availability during peak seasons (χ²=12.4, p=0.002) | **Full convergence** | Retained as positive (+) link |
| **LINK: Coffee yield → Child school attendance (+)** | Coffee-honey only | Mentioned in FGD #2 only (2 participants); KII #7 (1 participant) – insufficient across data sources | Survey showed no significant correlation (r=0.08, p=0.32; n=125) | **Divergence** | **Excluded** (insufficient evidence) |
| **LINK: Wealth status → Risk tolerance (+)** | All four systems | Mentioned in 2 FGDs (6 participants) but direction inconsistent (some said richer take more risks, others less) | No statistically significant difference in risk-taking behavior across wealth tertiles (χ²=3.4, p=0.18) | **Divergence** | **Excluded** (inconsistent evidence) |
| **LINK: Family size → Land productivity (-)** | Subsistence only | Mentioned in FGD #1 (4 participants) – weak support | Correlation between family size and yield per hectare: r=0.09 (p=0.21); no clear relationship | **Partial convergence** | Retained with caveat (weak link; documented in audit trail) |

**Table S3: Summary of Triangulation Outcomes by Farming System**

| Farming System | Total Variables/Links | Full Convergence | Partial Convergence | Divergence (Excluded) | Retention Rate |
| --- | --- | --- | --- | --- | --- |
| Subsistence | 24 | 19 (79%) | 3 (13%) | 2 (8%) | 92% |
| Coffee-honey | 28 | 23 (82%) | 3 (11%) | 2 (7%) | 93% |
| Maize-livestock | 26 | 21 (81%) | 3 (12%) | 2 (8%) | 92% |
| Wheat-livestock | 25 | 20 (80%) | 3 (12%) | 2 (8%) | 92% |
| **Total** | **103** | **83 (81%)** | **12 (12%)** | **8 (8%)** | **92%** |

**Legend for Convergence Status**

| Status | Definition | Action |
| --- | --- | --- |
| **Full convergence** | Qualitative evidence (≥3 participants, ≥2 data sources) and quantitative evidence (statistically significant or descriptively consistent) agree in direction and magnitude | Retain in final CLD |
| **Partial convergence** | One source strongly supports; other source is neutral, weakly supportive, or has insufficient data | Retain with caveat; document in audit trail |
| **Divergence** | Qualitative and quantitative evidence contradict, or one source strongly contradicts the other | Exclude from final CLD; document reason in audit trail |
