# Supplementary material for: Farming system dynamics and sustainability pathways in three districts of Jimma zone, Southwestern Ethiopia
Source: PLoS One. 2026 Oct 28;21(10):e0359541. doi: 10.1371/journal.pone.0359541 (PMC13716233; doi:10.1371/journal.pone.0359541)
Supplement: Supplementary file 3 [file pone.0359541.s003.docx]

**Supplementary File S3: Semi‑Structured Interview Guide**

**For Key Informant Interviews (KIIs) and Focus Group Discussions (FGDs)**

Study Title: Systems Dynamics Analysis of Interlinked Farming Systems and Sustainability Pathways in the Jimma Zone, Southwestern Ethiopia

Research Team: Tamiru Chalchisa (Principal Investigator), Abayneh Amare, Teferi Tolera, Meskelu Daniel, Dessalegn Obsi Gemeda

Data Collection Period**:** January – June 2025

Languages: Prepared in English, translated into Afan Oromo for fieldwork, back‑translated for accuracy.

**1. Introduction**

Good morning/afternoon. Thank you for agreeing to participate in this discussion/interview. My name is [______], and I am part of a research team from Jimma University. We are studying how different farming systems work, what challenges farmers face, and what can be done to make farming more sustainable in the face of climate change. Your experience and knowledge are very valuable. This discussion will take about 60–90 minutes. Everything you say will be kept confidential. You can refuse to answer any question or stop at any time. Do you have any questions before we begin?

**2. Interview / FGD Structure**

| Domain | Purpose | Questions | Probes |
| --- | --- | --- | --- |
| **A. Farming system characterisation** | Understand what farmers grow, raise, and how they manage their farms. | - What crops do you grow and what animals do you keep? - How much of what you produce do you sell vs. keep for home? - What technologies or practices do you use (e.g., irrigation, improved seeds, fertilisers)? | - Why do you choose these crops/animals? - Has this changed in the last 10 years? How? - What determines whether you plant a particular crop? |
| **B. Climate change perceptions** | Capture how farmers observe and interpret changes in weather and seasons. | - Have you noticed changes in rainfall, temperature, or seasons over the past 10–20 years? - How do these changes affect your crops, livestock, and daily work? - Do you receive any climate information? From where? Is it useful? | - When did you first notice these changes? - Can you give a specific example of a bad year? - What do you do when the rains are late or too heavy? |
| **C. Challenges and interlinked constraints** | Identify feedback loops, trade‑offs, and system‑specific traps. | - What are the biggest problems you face in farming? - How do problems in one part of your farm (e.g., soil, labour, money) affect other parts? - Do you see conflicts or competition between different types of farmers (e.g., coffee growers vs. livestock keepers)? - For subsistence farmers: What happens when food is scarce? How does that affect your work ability? - For coffee‑honey farmers: What happens to bees and honey when forest is cleared? - For maize‑livestock farmers: Where do you get animal feed in the dry season? What happens when you use maize stalks for cooking? - For wheat‑livestock farmers: Who does the weeding and harvesting? Is labour ever a problem? | - Can you describe a cycle that keeps you poor or trapped? - What would break that cycle? - How do you cope when fertiliser or seed is not available? - Do you borrow money? From whom? Is it easy or hard? |
| **D. Adaptation pathways and policy recommendations** | Explore what works, what farmers want, and what policies could help. | - What changes have you made to adapt to climate change? - What kinds of support would help you most (e.g., training, credit, better seeds, roads, storage)? - If you could change one thing about government or NGO programmes, what would it be? - For subsistence farmers: Would nutrition support or food aid help you work more? - For coffee‑honey farmers: Would you prefer more shade trees or more open coffee fields? Why? - For maize‑livestock farmers: Would you use biogas if it were available? Why or why not? - For wheat‑livestock farmers: Would you hire a tractor for planting? What stops you? | - What would make you invest more in your farm? - Have you heard of any new technologies you would like to try? - What stops you from using them? - What should the government do first? |

3. Additional Probe Questions for Specific Groups

- Why do some farmers in this village use [irrigation / improved seeds / agroforestry] while others do not?
- What do you learn from neighbors who are doing better?

**For female participants**

- Do women and men make different decisions about what to grow or sell? Who controls the income?
- What challenges do women face in getting water, credit, or training?

**For key informants (extension agents, scheme managers, administrators)**

- What are the main policies or programmes promoting sustainable farming in this district? Are they working?
- How do you prioritise which farmers to assist?
- What is the biggest gap between what farmers need and what your office can provide?

**4. Closing**

Thank you very much for your time and for sharing your experiences. Your answers will help us understand how to make farming more resilient and sustainable. Do you have any final questions or anything else you would like to add? If you think of something later, you can contact [name and phone number of local contact]. We will share the results with the kebele office when the study is finished.

**5. Instructions for Facilitators**

- Recording: Always ask permission before audio‑recording. If denied, take detailed written notes.
- Language: Conduct the discussion in Afan Oromo. Use simple, everyday words. Avoid technical jargon.
- Group management: For FGDs, ensure that no single participant dominates. Gently invite quieter participants. Keep the group size to 8–10.
- Note‑taking: Have a second researcher take notes on body language, group dynamics, and major themes. Write down verbatim quotes that sound particularly insightful.
- Ethics: Remind participants that there are no right or wrong answers. Respect privacy and stop if any participant becomes distressed.
- Duration: Stick to 60–90 minutes. If the group is still engaged, you may extend to 100 minutes but always ask if participants want to continue.
