# Supplementary material for: Farming system dynamics and sustainability pathways in three districts of Jimma zone, Southwestern Ethiopia
Source: PLoS One. 2026 Oct 28;21(10):e0359541. doi: 10.1371/journal.pone.0359541 (PMC13716233; doi:10.1371/journal.pone.0359541)
Supplement: Supplementary file 4 [file pone.0359541.s004.docx]

## Supplementary File S4: Compliance with CROSS and COREQ Guidelines

## A. Compliance with CROSS Guidelines (Quantitative Survey)

The CROSS guideline contains 19 sections with 40 items covering title, abstract, introduction, methods, results, and discussion (Sharma et al., 2021). Our quantitative survey (household survey of n=375) has been reported in full alignment with these items. The table below shows where each CROSS item is addressed in our revised manuscript.

**Table A: CROSS Checklist – Quantitative Survey Reporting**

| CROSS Section | CROSS Item | Location in Manuscript (Page/Line) | Brief Explanation |
| --- | --- | --- | --- |
| Title and abstract | Identify the study as a survey | Title, Abstract | Title specifies “Systems Dynamics Analysis … in the Jimma Zone”; abstract mentions “household survey of 375 randomly selected households”. |
| Introduction | Background and objectives | Page 5–7, lines 12–45 | Clearly states the research gap, justifies the need for a multi‑system survey. |
| Methods | Study design | Page 9, lines 15–25 (Section 2.2) | Described as “convergent mixed‑methods design with a cross‑sectional household survey.” |
| Methods | Sampling strategy | Page 10–11 (Section 2.3, Stages 1–4) | Multi‑stage stratified random sampling fully described. |
| Methods | Sample size calculation | Page 11 (Section 2.4) | Kothari formula provided; power analysis added. |
| Methods | Survey instrument | Page 12 (Section 2.3) | Questionnaire development, pre‑testing, and cultural adaptation described. |
| Methods | Data collection procedures | Page 12–13 | Face‑to‑face administration by trained enumerators in Afan Oromo. |
| Methods | Recruitment and consent | Page 14 (Section 2.5, 2.6) | Informed consent obtained; ethical approval stated. |
| Methods | Variables and measurement | Table 1 (Page 5–6) | Operationalisation of SES framework into measurable variables. |
| Results | Participant flow | Table 2 (Page 10–11) | Detailed sampling frame by district, kebele, and user status. |
| Results | Descriptive characteristics | Table 8 and 9 (Page 22–23) | Socio‑demographic and economic characteristics fully presented. |
| Results | Main results | Tables 10–13, Figures 4–7 | Causal loop diagrams and summary tables for each system. |
| Discussion | Key findings and interpretation | Section 4 (Page 32–39) | Each system’s findings interpreted with literature. |
| Discussion | Limitations | Section 5.5 (Page 39) | A dedicated limitations section (cross‑sectional, gender bias, etc.). |
| Discussion | Generalisability | Section 5.6 (Page 39) | Future research directions include comparative studies in other zones. |
| Other | Ethics and consent | Section 2.5 and 2.6 (Page 14) | IRB approval number, informed consent, confidentiality. |
| Other | Data availability | Page 40 (Section “Availability of data and materials”) | Statement provided. |
| Other | Funding | Page 40 (Section “Funding”) | Acknowledged. |
| Other | Conflicts of interest | Not applicable | Declared. |

## B. Compliance with COREQ Guidelines (Qualitative Methods)

The COREQ checklist contains 32 items across three domains: (1) research team and reflexivity, (2) study design, and (3) analysis and findings (Tong et al., 2007). Our qualitative component (8 FGDs, 21 KIIs, participant validation) is reported in accordance with these items.

**Table B: COREQ Checklist – Qualitative Methods Reporting**

| COREQ Domain | COREQ Item | Location in Manuscript (Page/Line) | Brief Explanation |
| --- | --- | --- | --- |
| **1. Research team and reflexivity** | | | |
| … 1.1 –1.3 | Personal characteristics (interviewer/facilitator) | Page 9 (Section 2.2) and Page 11 (Section 2.3) | Two trained researchers (PhD candidates) facilitated FGDs; one of them is the corresponding author with experience in the study area. |
| … 1.4–1.6 | Relationship with participants | Page 14 (Section 2.6) | Established trust through local development agents; no prior relationship disclosed. |
| **2. Study design** |  |  |  |
| … 2.1–2.2 | Theoretical framework | Page 4–5 (Section “Conceptual framework”) | Social‑Ecological Systems (Ostrom, 2009) framed the study. |
| … 2.3–2.5 | Participant selection | Page 10 (Section 2.3, Stage 2) | Purposive sampling by agro‑ecology, farming system, wealth status, gender. |
| … 2.6–2.9 | Setting and data collection | Page 11–13 (Section 2.3 and 2.4) | FGDs and KIIs conducted in Afan Oromo; audio‑recorded, transcribed verbatim; saturation protocol described. |
| … 2.10–2.13 | Data analysis | Page 12–13 (Section 2.4, Phases 1–3) | Thematic analysis using NVivo 14; step‑by‑step coding, inclusion criteria, causal link extraction. |
| **3. Analysis and findings** | | | |
| … 3.1–3.3 | Coding and theme development | Page 12–14 (Section 2.4) | Open coding by two independent researchers; inter‑coder agreement >80%; audit trail kept. |
| … 3.4–3.7 | Triangulation and validation | Page 14 (Section 2.4.1) and Table S1, S2 | Triangulation matrix (full convergence 81%); member checking (n=24 participants); expert validation (n=5). |
| … 3.8–3.12 | Use of participant quotes | Pages 25–30 (Section 5.1 and 5.2) | Anonymous quotes (FGD#, Participant#) provided for each system to illustrate findings. |

Tong, A., Sainsbury, P. and Craig, J. (2007) Consolidated criteria for reporting qualitative research (COREQ): a 32‑item checklist for interviews and focus groups. International Journal for Quality in Health Care, 19(6), pp. 349–357.
